# Allosteric regulation and crystallographic fragment screening of SARS-CoV-2 NSP15 endoribonuclease

**DOI:** 10.1093/nar/gkad314

**Published:** 2023-04-28

**Authors:** Andre Schutzer Godoy, Aline Minalli Nakamura, Alice Douangamath, Yun Song, Gabriela Dias Noske, Victor Oliveira Gawriljuk, Rafaela Sachetto Fernandes, Humberto D Muniz Pereira, Ketllyn Irene Zagato Oliveira, Daren Fearon, Alexandre Dias, Tobias Krojer, Michael Fairhead, Alisa Powell, Louise Dunnet, Jose Brandao-Neto, Rachael Skyner, Rod Chalk, Dávid Bajusz, Miklós Bege, Anikó Borbás, György Miklós Keserű, Frank von Delft, Glaucius Oliva

**Affiliations:** Sao Carlos Institute of Physics, University of Sao Paulo, Av. Joao Dagnone, 1100 - Jardim Santa Angelina, Sao Carlos, 13563-120, Brazil; Sao Carlos Institute of Physics, University of Sao Paulo, Av. Joao Dagnone, 1100 - Jardim Santa Angelina, Sao Carlos, 13563-120, Brazil; Diamond Light Source Ltd, Harwell Science and Innovation Campus, Didcot OX11 0QX, UK; Research Complex at Harwell, Harwell Science and Innovation Campus, Didcot OX11 0FA, UK; Electron Bio-imaging Centre, Diamond Light Source Ltd., Harwell Science and Innovation Campus, Didcot OX11 0QX, UK; Sao Carlos Institute of Physics, University of Sao Paulo, Av. Joao Dagnone, 1100 - Jardim Santa Angelina, Sao Carlos, 13563-120, Brazil; Sao Carlos Institute of Physics, University of Sao Paulo, Av. Joao Dagnone, 1100 - Jardim Santa Angelina, Sao Carlos, 13563-120, Brazil; Sao Carlos Institute of Physics, University of Sao Paulo, Av. Joao Dagnone, 1100 - Jardim Santa Angelina, Sao Carlos, 13563-120, Brazil; Sao Carlos Institute of Physics, University of Sao Paulo, Av. Joao Dagnone, 1100 - Jardim Santa Angelina, Sao Carlos, 13563-120, Brazil; Sao Carlos Institute of Physics, University of Sao Paulo, Av. Joao Dagnone, 1100 - Jardim Santa Angelina, Sao Carlos, 13563-120, Brazil; Diamond Light Source Ltd, Harwell Science and Innovation Campus, Didcot OX11 0QX, UK; Research Complex at Harwell, Harwell Science and Innovation Campus, Didcot OX11 0FA, UK; Diamond Light Source Ltd, Harwell Science and Innovation Campus, Didcot OX11 0QX, UK; Research Complex at Harwell, Harwell Science and Innovation Campus, Didcot OX11 0FA, UK; BioMAX, MAX IV Laboratory, Fotongatan 2, Lund 224 84, Sweden; Centre for Medicines Discovery, Oxford University, Oxford OX1 3QU, UK; Diamond Light Source Ltd, Harwell Science and Innovation Campus, Didcot OX11 0QX, UK; Research Complex at Harwell, Harwell Science and Innovation Campus, Didcot OX11 0FA, UK; Diamond Light Source Ltd, Harwell Science and Innovation Campus, Didcot OX11 0QX, UK; Research Complex at Harwell, Harwell Science and Innovation Campus, Didcot OX11 0FA, UK; Diamond Light Source Ltd, Harwell Science and Innovation Campus, Didcot OX11 0QX, UK; Research Complex at Harwell, Harwell Science and Innovation Campus, Didcot OX11 0FA, UK; Diamond Light Source Ltd, Harwell Science and Innovation Campus, Didcot OX11 0QX, UK; Research Complex at Harwell, Harwell Science and Innovation Campus, Didcot OX11 0FA, UK; Centre for Medicines Discovery, Oxford University, Oxford OX1 3QU, UK; Medicinal Chemistry Research Group, Research Centre for Natural Sciences, Magyar tudósok krt. 2, 1117 Budapest, Hungary; Department of Pharmaceutical Chemistry, University of Debrecen, Egyetem tér 1, 4032 Debrecen, Hungary; MTA-DE Molecular Recognition and Interaction Research Group, University of Debrecen, Egyetem tér 1, 4032 Debrecen, Hungary; Department of Pharmaceutical Chemistry, University of Debrecen, Egyetem tér 1, 4032 Debrecen, Hungary; National Laboratory of Virology, University of Pécs, Ifjúság útja 20, H-7624 Pécs, Hungary; Medicinal Chemistry Research Group, Research Centre for Natural Sciences, Magyar tudósok krt. 2, 1117 Budapest, Hungary; Diamond Light Source Ltd, Harwell Science and Innovation Campus, Didcot OX11 0QX, UK; Research Complex at Harwell, Harwell Science and Innovation Campus, Didcot OX11 0FA, UK; Centre for Medicines Discovery, Oxford University, Oxford OX1 3QU, UK; Department of Biochemistry, University of Johannesburg, PO Box 524, Auckland Park 2006, South Africa; Sao Carlos Institute of Physics, University of Sao Paulo, Av. Joao Dagnone, 1100 - Jardim Santa Angelina, Sao Carlos, 13563-120, Brazil

## Abstract

Severe acute respiratory syndrome coronavirus 2 (SARS-CoV-2) is the causative agent of coronavirus disease 2019 (COVID-19). The NSP15 endoribonuclease enzyme, known as NendoU, is highly conserved and plays a critical role in the ability of the virus to evade the immune system. NendoU is a promising target for the development of new antiviral drugs. However, the complexity of the enzyme's structure and kinetics, along with the broad range of recognition sequences and lack of structural complexes, hampers the development of inhibitors. Here, we performed enzymatic characterization of NendoU in its monomeric and hexameric form, showing that hexamers are allosteric enzymes with a positive cooperative index, and with no influence of manganese on enzymatic activity. Through combining cryo-electron microscopy at different pHs, X-ray crystallography and biochemical and structural analysis, we showed that NendoU can shift between open and closed forms, which probably correspond to active and inactive states, respectively. We also explored the possibility of NendoU assembling into larger supramolecular structures and proposed a mechanism for allosteric regulation. In addition, we conducted a large fragment screening campaign against NendoU and identified several new allosteric sites that could be targeted for the development of new inhibitors. Overall, our findings provide insights into the complex structure and function of NendoU and offer new opportunities for the development of inhibitors.

## Graphical Abstract

**Graphical Abstract:**
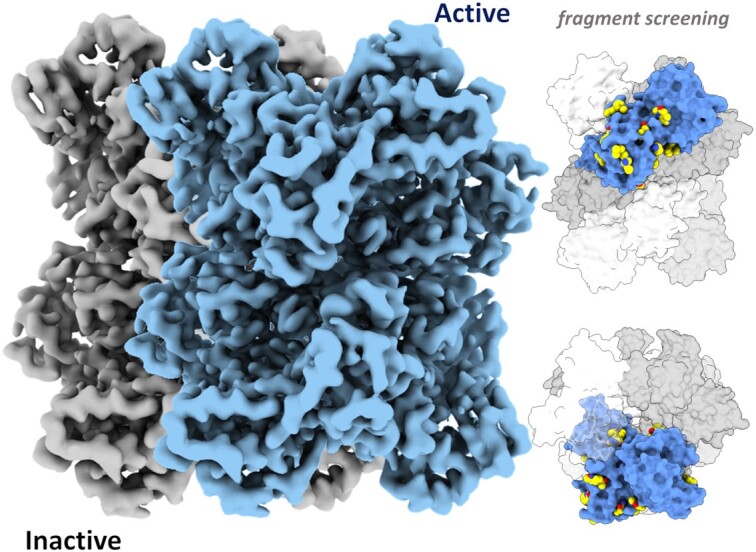
SARS-CoV-2 NendoU shifts between active and inactive conformation, and fragment screening revealed multiple allosteric sites.

## INTRODUCTION

The coronavirus disease 2019 (COVID-19) pandemic, caused by severe acute respiratory syndrome coronavirus 2 (SARS-CoV-2), has become a major global health and economic crisis (1, 2). The rapid and global dissemination of COVID-19 has resulted in an urgent need for the development of effective therapeutic options against this novel coronavirus (3). SARS-CoV-2 is a member of the *Betacoronavirus* genus, which includes SARS-CoV and MERS-CoV (1, 4). Its genome of ∼30 kb consists of a long replicase gene encoding non-structural proteins (NSPs), followed by structural and accessory genes (5). Due to a ribosomal frameshifting, the replicase gene encodes two open reading frames (ORFs), rep1a and rep1b, that are translated into two large polyproteins, pp1a and pp1ab (6). The cleavage of these results in 16 NSPs with distinct functions, including several replicative enzymes which are key targets for antiviral development (7).

One of the least understood NSPs of coronaviruses is NSP15, a highly conserved nidoviral RNA uridylate‐specific endoribonuclease (NendoU), carrying the catalytic domain of the endonuclease family (8, 9). NendoU is a three-domain 34 kDa protein that was demonstrated to form a barrel-shaped hexamer in solution by the assembly of two trimers (8, 10). The oligomerization is mostly driven by the contacts of the N-terminal (ND) and the middle (MD) domains, while the C-terminal domain (CD) carries the canonical catalytic residues from the endonuclease family (11). In the oligomer, the six active sites seem to be independently located at the top and bottom of the complex (Supplementary Figure S5). Despite NendoU showing maximum activity as an oligomer (12), the exact role of the hexamer is still unknown.

Due its unique activity, as well as the fact that this enzyme was co-localized with the *de novo* synthesized viral RNA (13) and the viral RNA-dependent RNA polymerase (NSP12) (14), a key protein of the viral replication machinery (15), it was suggested that NendoU is directly involved in viral RNA metabolism. Moreover, evidence suggests that NendoU is crucial for the innate immune evasion of coronaviruses due its antagonistic effects on interferon, promoted by the decreasing host cell double-stranded RNA (dsRNA) sensors, which was observed in macrophages and *in vivo* (16–20). Still, the exact role of NendoU in viral metabolism remains unclear, but accumulated evidence indicates that NendoU is a key viral enzyme and therefore a valuable target for antiviral development or even attenuated viral vaccines (17, 21). Still, the complex RNA binding mode of NendoU, lack of structural complexes and allosteric behavior hamper the development of new antivirals.

Here, we combine X-ray crystallography, cryo-electron microscopy (cryo-EM) and biochemical analysis to investigate the complex biochemical profile of SARS-CoV-2 NendoU in both monomeric (NendoU^mon^) and hexameric (NendoU^hex^) forms. Combined structural and biochemical analyses indicate that NendoU activity depends on the hexamer dynamics and suggest that supramolecular interactions are involved in the enzyme regulation mechanism. Furthermore, we carried out a large-scale X-ray fragment screen to probe new putative druggable sites. Our data shed light on the understanding of NendoU enzymatic profiles and opens up the path for the development of new allosteric and competitive inhibitors.

## MATERIALS AND METHODS

### Protein production and purification

The SARS-COV-2 cDNA sample was generously donated by Dr Edilson Durigon (GenBank MT126808.1), which was generated using SCRIPT One-Step RT-PCR (Cellco Biotec) and random hexamer primers. The NSP15 coding sequence was amplified using FastPol (Cellco Biotec) with primers 5′-CAGGGCGCCATGAGTTTAGAAAATGTGGCTTTTAATG-3′ and 5′-GACCCGACGCGGTTATTGTAATTTTGGGTAAAATGTTTCTAC-3′, and inserted into pETM11/LIC (generously donated by Dr Arie Geerlof) using the Ligation-Independent Cloning method (22). The final construct pETM11-NSP15 included an N-terminal 6×His-tag followed by a tobacco etch virus (TEV) cleavage site and residues 1–346 of NSP15.

Transformed BL21(DE3) cells were growth in LB Lennox medium supplemented with kanamycin at 37°C until OD_600_ = 1.0 and then kept for 16 h at 18°C after induction with 0.5 mM isopropyl-β-d-1-thiogalactopyranoside. Harvested cells were resuspended in Buffer A (50 mM Tris–HCl pH 8.0, 300 mM NaCl, 10% glycerol, 20 mM imidazole) supplemented with 0.1 mg/ml lysozyme, 10 U of benzonase (Cellco Biotec) and 1.0 mM dithiothreitol (DTT), and disrupted using sonication. Cleared lysate was passed through a 5 ml HisTrap HP column (GE Healthcare) equilibrated in Buffer A, and them protein was eluted with Buffer A supplemented with 300 mM imidazole. Excess imidazole was removed using a Sephadex XK 26/60 column (GE Healthcare) equilibrated with Buffer A. To obtain NSP15 in monomeric form (NendoU^mon^), the sample was incubated overnight at 8°C with 4 mM DTT and 0.1 mg/ml TEV protease, then passed through a 5 ml HisTrap HP column equilibrated in Buffer A to remove non-cleaved protein and TEV protease. To obtain NSP15 in hexameric form (NendoU^hex^), the 6×His-tag was not cleaved. For both forms, final purification was performed by size exclusion chromatography using a HiLoad Superdex 75 16/60 column (GE Healthcare) equilibrated with 20 mM HEPES pH 7.5, 150 mM NaCl, 5% (v/v) glycerol and 0.5 mM TCEP. Purity was confirmed by 12% sodium dodecylsulfate–polyacrylamide gel electrophoresis (SDS–PAGE). Protein concentration was estimated using the theoretical extinction coefficient at 280 nm of 33 140/M/cm (23). Mutations were generated by the inverse-polymerase chain reaction (PCR) method.

### Endoribonuclease assay and kinetic analysis

Fluorogenic oligonucleotide substrate 5'6-FAM-dArUdAdA-6-TAMRA3' was purchased from GenScript. Unless stated otherwise, activity assays were performed in 25 mM Bis-Tris–HCl buffer pH 6.0, 0.25 μM substrate and 100 nM NendoU^mon^ or 25 nM NendoU^hex^ at 35°C. Time-course reactions were directly monitored in a Stratagene Mx3005P real-time PCR system (Agilent Technologies) using FAM filters. The initial velocity (v_0_) was estimated using the slope from linear regressions of the reaction monitored during minutes 3–10. All reactions were performed in triplicate, and reported data are the average and error bars the standard deviation (SD). Analysis and graphs were performed with OriginPro v9.0.

The gel-based RNA cleavage assay was performed to verify the cleavage of the poly(U) biotinylated RNA substrate 5'-UGACCUCUAGCUAGAGGUCA(U)_30_-3' by nsp15, based on (24). A 15% acrylamide/urea gel (8 M) was pre-run at 200 V for 30 min in TBE buffer. Samples in 1× formamide loading buffer (Gel loading buffer II, Ambion) were heated for 5 min at 95°C prior to analysis. The denaturing gel was run for 2 h at 200 V and RNA bands were visualized with SYBR Safe DNA Gel Stain (Invitrogen) under UV light.

### Cryo-EM sample preparation and data collection

For sample preparation, 3 μl of 0.5 mg/ml NendoU^hex^ in experimental buffer were applied on the Quantifoil 300 mesh copper R1.2/1.3 grid. Buffers tested were: 50 mM Tris–HCl pH 7.5, 200 mM NaCl; 50 mM Bis-Tris–HCl pH 6.0, 200 mM NaCl; or 50 mM phosphate-buffered saline (PBS) pH 6.0, 200 mM NaCl. Samples containing oligo(dT) were made in 50 mM PBS pH 6.0, 200 mM NaCl buffer containing 5 μM oligo(dT)_20_ (Thermofisher™). The sample was blotted for 4 s (4°C, 100% humidity) with a blot force of –12 and then plunge-frozen using a Vitrobot Mark IV (Thermo Fisher). Frozen samples were imaged on a Titan Krios (G2) microscope operated at 300 kV. Movies were collected on a K3 detector in super resolution counting mode with a slit width of 20 eV and at a nominal magnification of 105kx corresponding to a calibrated physical pixel size of 0.831 Å at the specimen level. Data acquisition was done using EPU software (version 2.6) with a defocus range of 1.4–3.2 nm. Data collection statistics are summarized in Supplementary Table S2.

### Cryo-EM data processing and modeling

In summary, all movies were aligned using MotionCor2 (25), and processed using cryoSPARC v2.15 (26). Contrast transfer function (CTF) parameters for each micrograph were determined by Patch CTF. Initial templates were constructed using manual picking tools, and then all micrographs were picked using Template Picker, followed by extraction with a box size of 256 px and two rounds of 2D classification. Selected particles were used for *ab initio* modeling and two rounds of heterogeneous refinement. Selected volumes were refined using Non-uniform Refinement and D3 symmetry. The detailed data processing flowchart schemes for samples in buffers 50 mM Tris–HCl pH 7.5 mM, 200 mM NaCl; 50 mM Bis-Tris–HCl pH 6.0, 200 mM NaCl; or 50 mM PBS pH 6.0, 200 mM NaCl are available in Supplementary Figures S6, S7 and S8, respectively, and final gold standard Fourier shell correlation (FSC) resolution for the models were2.98, 3.2 and 2.5 Å, respectively. For modeling, ChimeraX was used for rigid fitting of the model using PDB 7KF4 as template, followed by real space refinement using Phenix (27, 28). Data collections and model refinement statistics are summarized in Supplementary Table S2. To analyze variability of SARS-CoV-2 NSP15 structural flexibility, we used the 3D Variability Analysis (3DVA) tool available in cryoSPARC v2.15 (29). Particles and a mask were used to compute three eigenvectors of the covariance matrix of the data distribution using 3DVA (29), with resolution filtered at 4 Å. Model series were generated using the 3DVA display tool. Movies were generated with ChimeraX v1.2.5.

### Helical processing for NSP15 filaments

Helix processing was performed with relion v3.1 (30). Data were aligned with MotionCorrv2 (25), and CTF was corrected with ctffind v4.1.13 (31). Particle picking was performed manually with the relion manual helical picking tool using a 120 Å particle diameter. Particles were extracted (2437) with box of 600 pixels as helical segments (tube diameter 150 Å and start–end mode, helical rise 10, number of asymmetry units 6). Particles were classified with Class2D as Helix segments, with 150 Å tube diameter, bimodal angular search, angular search psi 6° and helical rise 10 Å.

### Crystallization, data collection and data processing of NendoU X-ray models

Crystals of NendoU^mon^ were obtained in multiple conditions. For that, the protein was concentrated to 3.4 mg/ml and crystallized in a sitting drop at 18°C by vapor diffusion. Hexagonal crystals containing a dihedral asymmetric unit were obtained in 15% polyethylene glycol (PEG) 8000, 0.1 M sodium/potassium phosphate pH 6.2 and 20% (w/v) PEG 3350, 100 mM Bis-Tris propane, pH 6.5, 200 mM sodium sulfate, and cryo-conditions were obtained by adding 20% (v/v) ethylene glycol. Orthorombic crystals containing a hexagonal asymmetric unit were obtained with protein incubated with 100 μM oligo(dT) and crystallized in 0.1 M trisodium citrate pH 5, 14% (w/v) PEG 6000, and cryo-conditions were obtained by adding 20% (v/v) ethylene glycol.

Data were processed with XDS via autoPROC (32, 33), scaled with Aimless via CCP4 (34), solved by molecular replacement using PDB 6X1b as template via Phaser (35) and refined with phenix.refine or BUSTER (36, 37). The model was built with Coot and validated with Molprobity (38, 39) Projection of electrostatic potential into the surface was calculated with APBS standard setups, and figures were generated using PyMOL and ChimeraX (28, 40). Data collection and refinement statistics are available in Supplementary Table S3. Conservation analyses were performed with Consurf (41), and the results are available in Supplementary Figure S13.

### Fragment screening of SARS-CoV-2 NendoU

For the fragment screening campaign, protein crystals were formed using 300 nl of NendoU^mon^ at 3.4 mg/ml mixed with 300 nl of well solution containing 14% (w/v) PEG 6000, 0.1 M tri-sodium citrate pH 5.0 and 10 nl of seed stocks of the same condition in Swissci 96-Well 3-Drop plates (Molecular Dimensions) containing 30 μl of well solution. Crystals were formed after 4 days at 20°C by vapor diffusion. For soaking, 40 nl of each fragment compound from the XCHEM Poised Library (42) and OPEN-EU DRIVE fragment library (final concentration of 100 mM) were added to a crystallization drop using an ECHO acoustic liquid handler dispenser at the Diamond light source XChem facility (43) Cryo-conditions were created by adding 20% ethylene glycol to the drops using an ECHO acoustic liquid handler. Crystals were soaked for 2 h before being harvested using XChem SHIFTER technology, and data were collected at the beamline I04-1 in automated mode. The XChem Explorer pipeline (44) was used for structure solution with parallel molecular replacement using DIMPLE (34). Fragments were identified using PANDDA software (45) and CLUSTER4X (46). Data were modeled and refined using phenix.refine, BUSTER and COOT (36–38), and validated using Molprobity (39). Coordinates and structure factors of the ground-state model were deposited in the PDB under the code 5SBF. Bound-state statistics and PDB codes are summarized in the Supplementary data analysis table (Supplementary Table 1).

### Native electrospray ionization-time of flight mass spectrometry (ESI-TOF MS) intact mass analysis

Proteins for native MS were held on ice and buffer exchanged into 75 μl of 50 mM ammonium acetate pH 7.5 by three rounds of gel filtration using BioGel P6 (Biorad) spin columns according to the manufacturer's instructions. Mass spectra were acquired using an Agilent 6530 QTOF operating in positive ion 1 GHz mode using a standard ESI source. Samples were introduced via a syringe pump at a flow rate of 6 μl/min. The ion source was operated with the capillary voltage at 3500 V, nebulizer pressure at 17 psig, drying gas at 325°C and drying gas flow rate at 5 l/min. The instrument ion optic voltages were as follows: fragmentor 430 V, skimmer 65 V and octopole RF 750 V. The *m/z* spectra were analyzed using Masshunter B.07.00 (Agilent); ESIprot (47) and employing an ion table.

### Chemical synthesis of nucleoside analogs

5′-Azido-5′-deoxy-thymidine (LIZA-7) was prepared according to the procedure we published recently (48) [the first synthesis of this compound was reported in 2007 (49)].

5′-Deoxy-5′-thiothymidine (FÜZS-5**)** was prepared according to a novel procedure reported here, through the deacetylation of 5′-*S*-acetyl-5′-deoxy-5′-thiothymidine, with the latter being synthesized based on the procedure reported by Kawai *et al.* (50) [the first synthesis of 5′-deoxy-5′-thiothymidine via the deacetylation of the 3′,5′-diacetyl analog was reported in 1964 (51)]. Reagents were purchased from Sigma-Aldrich Chemical Co. and used without further purification. Optical rotations were measured at room temperature with a Perkin-Elmer 241 automatic polarimeter. Thin-layer chromatography (TLC) was performed on Kieselgel 60 F_254_ (Merck) with detection by UV light (254 nm) and immersion into sulfuric acidic ammonium molybdate solution or 5% ethanolic sulfuric acid followed by heating. Flash column chromatography was performed on silica gel 60 (Merck, 0.040–0.063 mm). The ^1^H nuclear magnetic resonance (NMR) (400 MHz) and ^13^C NMR (100 MHz) spectra were recorded with a Bruker DRX-400 spectrometer at 25°C. Chemical shifts are referenced to Me_4_Si (0.00 ppm for ^1^H) and to the residual solvent signals (CDCl_3_, 77.2; DMSO-d_6_, 39.5; CD_3_OD, 49.0 for ^13^C). Matrix-assisted laser desorption/ionization-time of flight mass spectrometry (MALDI-TOF MS) analyses of the compounds was carried out in the positive reflectron mode using a BIFLEX III mass spectrometer (Bruker, Germany) equipped with delayed-ion extraction. 2,5-Dihydroxybenzoic acid (DHB) was used as the matrix and F_3_CCOONa as cationizing agent in dimethylformamide (DMF). ESI-TOF MS spectra were recorded by a microTOF-Q type QqTOFMS mass spectrometer (Bruker) in the positive ion mode using MeOH as the solvent.

The purity of compounds FÜZS-4, FÜZS-5 and LIZA-7 was assessed via high-perfomance liquid chromatography (HPLC)-MS measurements, using a Shimadzu LCMS-2020 device equipped with a Reprospher 100 C18-DE (5 μm; 100 × 3 mm) column, a Shimadzu SPD-M20A photodiode array detector and a positive–negative double ion source (DUIS±) with a quadrupole MS analyser in a range of 50–1000 *m/z*. Samples were eluted with an gradient elution using eluent A (0.1% formic acid in water) and eluent B (0.1% formic acid in acetonitrile). The flow rate was set to 1 ml/min. The initial condition was 0% B eluent, followed by a linear gradient to 100% B eluent by 2 min; from 2 to 3.75 min 100% B eluent was retained and then from 3.75 to 4.5 min it was returned to the initial condition and retained to 5 min. The column temperature was kept at 30°C and the injection volume was 8 μl. Following peak integration of the UV/VIS chromatograms, purities for all three compounds were determined to be >95%.

MALDI-TOF MS measurements were carried out with a Bruker Autoflex Speed mass spectrometer equipped with a TOF mass analyzer. In all cases, 19 kV (ion source voltage 1) and 16.65 kV (ion source voltage 2) were used. For reflectron mode, 21 kV and 9.55 kV were applied as reflector voltage 1 and reflector voltage 2, respectively. A solid phase laser (355 nm, ≥100 μJ/pulse) operating at 500 Hz was applied to produce laser desorption, and 3000 shots were summed. 2,5-Dihydroxybenzoic acid (DHB) was used as matrix and F3CCOONa as cationic agent in DMF.

**5′-*S*-Acetyl-5′-deoxy-5′-thiothymidine (Th-5′SAc, FUZ-4):** PPh_3_ (6.5 g, 24.6 mmol, 2.0 equiv.) was dissolved in dry tetrahydrofuran (THF; 50 ml) and cooled to 0°C. Diisopropyl azodicarboxylate (DIAD) (5.0 ml, 24.6 mmol, 2.0 equiv.) was added dropwise and stirred for 30 min at 0°C. Thymidine (3.0 g, 12.3 mmol) and HSAc (1.8 ml, 24.6 mmol, 2.0 equiv.) were dissolved in dry DMF (50 ml), added dropwise to the reaction mixture and stirred for 30 min at 0°C and 30 min at room temperature. The solvent was evaporated under reduced pressure and the crude product was purified by flash column chromatography (gradient elution, CH_2_Cl_2_/MeOH 97.5/2.5→95/5) to give **5′-*S*-acetyl-5′-deoxy-5′-thiothymidine** (1.7 g, 46%) as a yellowish solid.

R_f_ = 0.21 (CH_2_Cl_2_/MeOH 95/5), [α]_D_ = +40.9 (*c* 0.11, DMSO),^1^H NMR (400 MHz, DMSO) *δ* (ppm) 7.45 (d, *J* = 0.8 Hz, 1H, H-6), 6.16 (dd, *J* = 7.6, 6.5 Hz, 1H, H-1′), 5.43 (d, *J* = 4.3 Hz, 1H, O*H*), 4.11 (td, *J* = 6.7, 3.4 Hz, 1H, H-3′), 3.80–3.71 (m, 1H, H-4′), 3.23 (dd, *J* = 13.8, 5.8 Hz, 1H, H-5′a), 3.11 (dd, *J* = 13.8, 7.3 Hz, 1H, H-5′b), 2.37 (s, 3H, AcC*H*_3_), 2.28–2.18 (m, 1H, H-2’a), 2.06 (ddd, *J* = 13.5, 6.2, 3.3 Hz, 1H, H-2′b), 1.81 (s, 3H, thymine C*H*_3_). ^13^C NMR (100 MHz, DMSO) *δ* (ppm) 194.9 (1C, Ac*C*O), 163.7, 150.5 (2C, C-2, C-4), 136.1 (1C, C-6), 109.9 (1C, C-5), 84.5, 83.9, 72.6 (3C, C-1′, C-3′, C-4′), 38.0 (1C, C-2′), 31.1 (1C, C-5′), 30.6 (1C, Ac*C*H_3_), 12.2 (1C, thymine *C*H_3_). MALDI-ToF MS: *m/z* calculated for C_12_H_16_N_2_NaO_5_S ^+^ [M + Na]^+^ 323.0672, found 323.0660.

**5′-Deoxy-5′-thiothymidine (FÜZS-5):** 5′-*S*-acetyl-5′-deoxy-5′-thiothymidine (1.7 g, 5.7 mmol) was dissolved in dry MeOH (25 mL) under argon, and NaOMe (458 mg, 8.5 mmol, 1.5 equiv.) was added and stirred at room temperature for 4 h. The reaction mixture was neutralized with Amberlite IR 120 H^+^ ion exchange resin, filtered and evaporated under reduced pressure. The crude product was purified by flash column chromatography (CH_2_Cl_2_/MeOH 97.5/2.5→95/5) to give **5′-deoxy-5′-thiothymidine (FÜZS-5)** (826 mg, 59%) as a white solid.

R_f_ = 0.20 (CH_2_Cl_2_/MeOH 95/5), m.p. 200–202°C; [α]_D_ = +5.0 (*c* 0.28, DMSO), ^1^H NMR (400 MHz, DMSO) *δ* (ppm) 7.51 (s, 1H, H-6), 6.19 (t, *J* = 6.4 Hz, 1H, H-1′), 5.36 (s, 1H), 4.22 (s, 1H), 3.78 (t, *J* = 5.9 Hz, 1H), 2.75 (t, *J* = 7.1 Hz, 1H), 2.49 (d, *J* = 15.6 Hz, 1H), 2.21 (dt, *J* = 13.7, 6.9 Hz, 1H), 2.06 (dd, *J* = 11.8, 4.4 Hz, 1H), 1.80 (s, 3H, thymine C*H*_3_). ^13^C NMR (100 MHz, DMSO) *δ* (ppm)163.7150.5 (2C, C-2, C-4), 136.1 (1C, C-6), 109.9 (1C, C-5), 87.1 (1C, C-1′), 83.7 (1C, C-4′), 71.9 (1C, C-3′), 38.1 (1C, C-5′), 26.3 (1C, C-2′), 12.2 (1C, thymine *C*H_3_). MALDI-TOF MS: *m/z* calculated for C_10_H_14_N_2_NaO_4_S ^+^ [M + Na]^+^ 281.0566, found 281.0540.

## RESULTS AND DISCUSSION

### Recombinant NendoU can be obtained as monomers or hexamers

NendoU^mon^ and NendoU^hex^ were successfully purified as described. NendoU^mon^ was obtained after cleavage of the N-terminal histidine tag as a single monodisperse peak by gel filtration (Supplementary Figure S1). This diverges from previous reports, where NendoU was purified as a mix of monomers, trimers and hexamers under similar conditions (12, 52). However, these previous authors also reported that they had issues removing the N-terminal histidine tag from their constructs. A similar gel filtration profile was obtained when we kept the N-terminal histidine tag on the recombinant protein. This method shows that recombinant NendoU can be obtained mostly as hexamers by keeping the N-terminus, while cleaved NendoU is obtained mostly as monomers (Supplementary Figure S2). The importance of internal NSP15 N-terminal residues for proper oligomerization was already described for SARS-CoV NendoU, where the authors demonstrated that mutants such as E3A were exclusively expressed as monomers or trimers (12). Another possibility is that the N-terminal TEV site is only exposed in the monomeric form, while the hexamer offers some steric hindrance that blocks the cleavage. Nevertheless, mass spectroscopy confirms that monomeric samples are composed of cleaved protein while hexameric samples contain intact protein (Supplementary Figure S3). The native mass profiles of NendoU^mon^ and NendoU^hex^ showed that the former consists of the folded monomer as the principal species with higher order species as minor components, while NendoU^hex^ consists of the folded hexamer as the principal species with the folded monomer and dodecamer as less abundant species (Supplementary Figure S4). The native mass profile also indicates that uncleaved monomers have higher thermal stability than cleaved monomers, suggesting some sort of stabilization promoted by the N-terminal tag (Supplementary Figure S4). The two samples obtained were enzymatically characterized and used in structural studies to help us understand the function of oligomerization in NendoU activity and regulation.

### NendoU activity can be controlled by pH

The activities of NendoU^mon^ and NendoU^hex^ were tested in multiple buffers, at different pHs and salt concentrations, using a fluorogenic RNA oligo (53). For NendoU^mon^, significant activity was only observed in slightly acid buffers, such as sodium acetate pH 5.0 (v_0_ = 19.6 ± 0.12 RFU/min), sodium cacodylate pH 6.0, 150 mM NaCl (v_0_ = 15.5 ± 0.08 RFU/min), MES pH 6.0, 150 mM NaCl (v_0_ = 16.5 ± 0.07 RFU/min) and Bis-Tris pH 6.5, 150 mM NaCl (v_0_ = 15.6 ± 0.14 RFU/min). A similar behavior was seen for NendoU^hex^, that presented maximum activity at sodium cacodylate pH 6.0, 150 mM NaCl (v_0_ = 998.4 ± 31.2 RFU/min) and Bis-Tris pH 6.5, 150 mM NaCl (v_0_ = 902.8 ± 13.90 RFU/min). For more basic buffers, such as MOPS pH 7.0, HEPES pH 7.5 and Tris pH 8.5, the enzyme activity of both NendoU^mon^ and NendoU^hex^ was virtually negligible. In PBS with 150 mM NaCl, NendoU^hex^ activity was also significantly higher at pH 6.0 (v_0_ = 430.4 ± 15.0 RFU/min) than at pH 7.4 (v_0_ = 74.2 ± 3.0 RFU/min). Moreover, the overall reaction rate for NendoU^hex^ was much higher than for NendoU^mon^ for all tested conditions, similar to what has been reported previously (52, 12). NendoU^mon^ samples retain only ∼1–5% of the enzymatic activity of NendoU^hex^ in similar conditions, which could be correlated with contamination or spontaneous formation of larger oligomeric states in the sample, as suggested by Supplementary Figure S3A. In our assays, it was also possible to observe that NendoU^hex^ enzymatic activity is inversely proportional to salt concentrations, with a 3-fold increment of the activity in HEPES pH 7.5 in comparison with the same buffer added at 500 mM. Further studies were therefore conducted in 50 mM Bis-Tris buffer pH 6.0. All enzymatic activity values and details of the buffer are available in Supplementary Table S1.

### NendoU is an allosteric enzyme with a positive cooperativity index

**Figure 1.**
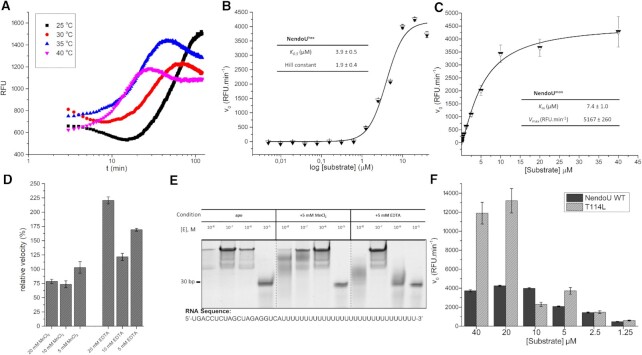
Summarized enzymatic profile characterization of NendoU. (**A**) Time-course reaction of NendoU^hex^ at different temperatures. (**B**) Calculated Hill constant for NendoU^hex^, showing a positive cooperative index of 2. (C) Michaelis–Menten plot of NendoU^mon^, showing a typical first-order enzymatic profile. (**D**) The relative enzymatic activity of NendoU^hex^ in thepresence of different concentrations of MnCl_2_ and EDTA. (**E**) Acrylamide gel showing the activity of NendoU^hex^ against poly(U) RNA in the presence of different concentrations of MnCl_2_ and EDTA. (**F**) Calculated initial velocities of mutant T114L and wild-type (WT) NendoU^hex^.

While the NendoU^mon^ time-course reaction exhibited the classical behavior of a first-order reaction, the NendoU^hex^ time-course reaction was clearly showing signs of a positive cooperative reaction, with a slow initial velocity that was seen to enter the exponential phase after 5 min (Figure 1A). We therefore tested selected temperatures to determine the time it took for NendoU^hex^ to reach halfmaximum activity at the exponential phase (*t*_1/2_). The *t*_1/2_ for temperatures of 25, 30, 35 and 40°C was 53.0, 29.7, 18.2 and 11.4 min, respectively (Figure 1A). The data show that higher temperatures influence not only the initial velocity, but also the positive cooperativity index of NendoU^hex^. It is also possible to observe a linear relationship in the 3–10 min interval for temperatures of 35 and 40°C, with an *R*^2^ of 0.96 and 0.97, respectively (Figure 1A). Therefore, all further analyses were performed at 35°C, and the initial velocity (v_0_) was estimated using the slope from linear regressions of the reaction monitored during minutes 3–10. Under these conditions, we were able to calculate the values of *K*_0.5_ 3.9 ± 0.5 μM for NendoU^hex^, and *K*_m_ of 7.4 ± 1.0 μM for NendoU^mon^, indicating that the hexamer form has an increased affinity for the substrate (Figure 1B, C). For NendoU^hex^, we also determined a Hill constant of 1.9 ± 0.4, indicating a positive collaboration between the sites of NSP15 (Figure 1B).

### NendoU is not controlled by manganese

In terms of substrate specificity, NendoU of the coronaviruses is described to cleave a great variety of single- and double-stranded RNAs in an Mn^2+^-dependent reaction, by hydrolyzing the 3′ end of pyrimidines, with a strong preference for uracil, and to release 2′,3′-cyclic phosphate and 5′-hydroxyl ends as products (11, 54, 55). However, despite the many studies claiming an enhanced activity of NendoU in the presence of Mn^2+^ (7, 54, 56), the ion was never observed to form a complex with the polymer or substrates and products (7, 54, 56). Furthermore, the structural comparison of the NendoU active site with well-characterized ion-independent endonucleases, such as *Bos taurus* RNase A, shows minor differences between the position of key catalytic residues (7, 11), and the ion was also never observed to complex with the crystal structures of NendoU. Notwithstanding, during our structural studies described below, we collected multiple fluorescence spectra from different crystals, but none of them indicated Mn in the samples (data not shown). Similarly, differential scanning fluorimetry showed no significant shift in enzyme stability in the presence or absence of any Mn ions (data not shown).

We also tested the enzymatic activity of NendoU^hex^ in the presence of several additives, including MnCl_2_ and MnSO_4_, and none of them showed a significant enhancement of the enzyme ratio. Next, we tested MnCl_2_ as an additive at various concentrations, and none of those was shown to enhance the enzyme activity (Figure 1D). Furthermore, we also tested the enzyme activity in high concentrations of EDTA, an obvious but missing control in most of above-mentioned studies, and the activity of NendoU^hex^ was not diminished for any of the tested concentrations, and was even enhanced in some cases (Figure 1D). To investigate its effect in a non-fluorescent assay, we performed an acrylamide/urea denaturating RNA PAGE analysis in reactions containing NendoU^hex^ and a synthetic poly(U) RNA oligo, with or without Mn and using EDTA as control. Manganese was not observed to be crucial for the RNA cleavage, and EDTA does not seem to have any deleterious effect on the reaction (Figure 1E). This seems also to be true for NendoU^mon^ sample, where we saw no significant change in the activity relative to control for samples containing MnCl_2_ or EDTA (Supplementary Table S4). We therefore found no evidence to show that NendoU is dependent on manganese or any other metal ions.

### NendoU binding to RNA involves the active site and switch regions

**Figure 2.**
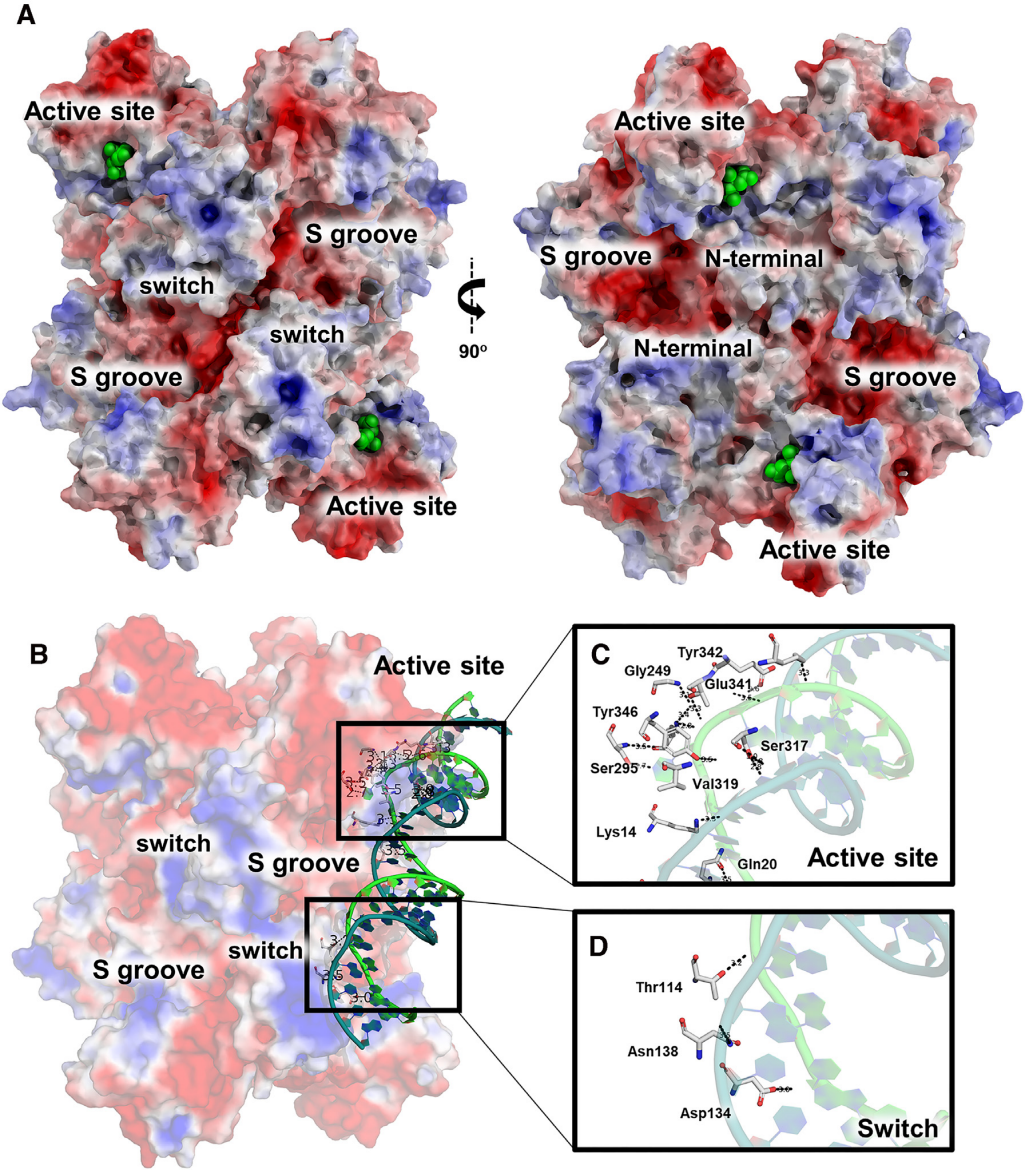
Overview of the NendoU structure. (**A**) Two distinct views of the NendoU hexamer surface (PDB 7KF4), highlighting regions of interest, including the active site, the S groove and the switch region. Citrate molecules on the active site are depicted as green spheres. (**B**) NendoU–dsRNA binding mode on the surface of the hexamer, occupying one active site and interacting with the switch region. (**C**) Detailed dsRNA binding mode on the active site of NendoU. (**D**) Detailed dsRNA binding mode on the switch region of NendoU. The structure of NendoU is colored according to its electrostatic potential projected on surface charge (–5 to 5 f kJ/mol/e in red-white-blue color model). dsRNA is colored in green/blue and was depicted from PDB 7TJ2.

At the middle surface of the barrel structure, we can find three pronounced cavities between each dimer subunit, which resemble the letter S (52) (Figure 2A). Due to their large size and proximity to the active site, it was assumed that these S cavities would serve to accommodate and recognize the variety of RNAs that can be processed by NendoU (11, 52). However, despite the many X-ray (8, 52, 56, 57) and cryo-EM (11) structures of NendoU of β-coronaviruses available to date, none of them shows any evidence of a nucleic acid or nucleosides bound to an S cavity. Moreover, we observed that electrostatic potential calculations of NendoU suggest a strong negatively charged surface for this cavity (Figure 2A), which is the opposite of what is expected for canonical DNA/RNA-binding protein recognition sites (58).

In fact, a recent cryo-EM study of NendoU with dsRNA shows that the RNA interacts not only with the active site, but also with the positively charged region (here named switch, an interface region comprising residues 96–121 from the MD) from an adjacent nsp15 chain (Figure 2B), but not with the S cavity (59). The structure revealed the binding mode of 5′ guanine π–π stacking with Trp332 (4.1 Å) at the +2 subsite, and the 3′ processed uridine base bound at the +1 site, showing an unusual inversion of the base moiety angles to accommodate the interactions of its O2 with His249 NE2 (3.1 Å) and O4 with Leu345 N (4.5 Å). These interactions are key to understanding not only the specificity of the uridine site for P1 uridine, but also its preference for purine bases at the +2 site (52, 56). This could also be observed in our experiment using the poly(U) synthetic RNA, where the cleavage shows that the 30 uracil tail is intact after the reaction, highlighting the importance of a +2 purine for proper cleavage (Figure 1E). This preference for purine bases 3′ of the cleaved uridine was also observed by Frazier *et al.* (60).

The binding of dsRNA to the NendoU surface also revealed that the interactions seem to be mediated by the positively charged residues of NendoU and the phosphate groups of the ribonucleosides (Figure 2C, D). The lack of any major interactions of sugar and base moieties with the enzyme, as well as the flattened disposition of these subsites, would explain the ability of NendoU in processing a broad variety of uridine-containing sequences (52, 12, 10). At the negative subsites, which are located at the top edge of the NendoU barrel, we also notice the absence of any obvious cavity that would recognize any base in a specific manner, agreeing with the observation that multiple 3′ extensions can be recognized and processed by the enzyme (52, 12, 10). We can also see that the enzyme surface of this area is strongly negatively charged, which would cause the rapid expulsion of any nucleic acid products after cleavage (Figure 2A). All this being considered, the specificity of NendoU seems to be restricted to the uridine site at +1, and the purine site at +2, narrowing our window for using structure-based methods for the development of competitive inhibitors.

### Cryo-EM reveals that NendoU switch conformation can be modulated by pH and determines open–closed conformations

**Figure 3.**
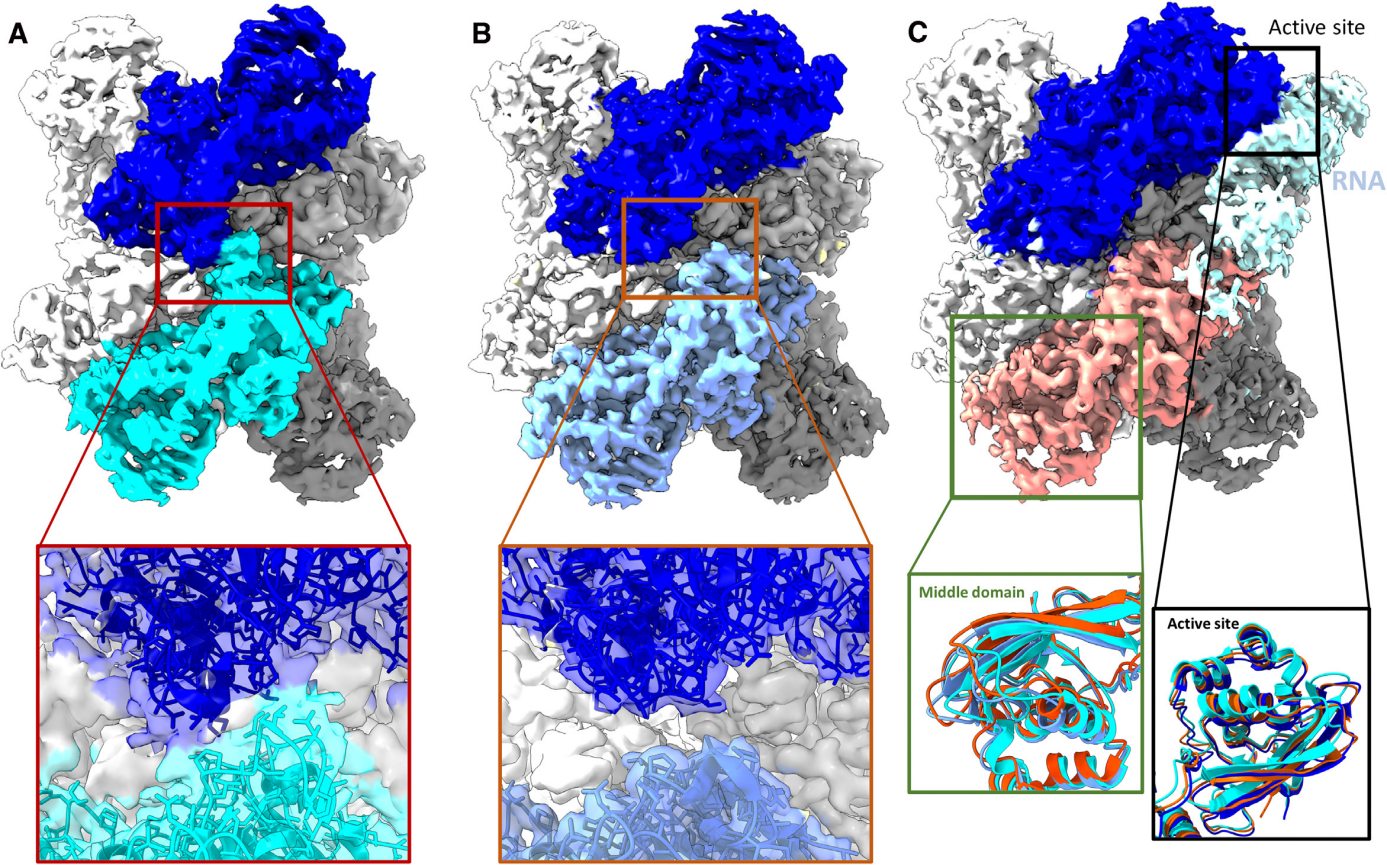
Cryo-EM maps of NendoU in different conditions. (**A**) Cryo-EM maps of NendoU in HEPES at pH 7.5 (PDB 7RB0) in the closed state, with chains A and B colored in blue and cyan, respectively. The box shows a detailed view of the map and model from the switch region between these two chains. (**B**) Cryo-EM maps of NendoU in PBS at pH 6.0 (PDB 7ME0) in the open state, with chains A and B colored in blue and light blue, respectively. The box shows a detailed view of the map and model from the switch region between these two chains. (**C**) Cryo-EM maps of NendoU in complex with dsRNA (PDB 7TJ2) and in the open state, with chains A and B colored in blue and salmon pink, respectively. THe dsRNA map is colored in light blue. The black box shows a detailed view of the three models aligned in the active site region. The green box shows detailed views of the middle domain of the three models as cartoons superposed on adjacent NendoU. 7RB0, 7ME0 and 7TJ2 are colored in dark blue, cyan and salmon pink, respectively.

During our cryo-EM studies, we were able to elucidate multiple high-quality models of NendoU^hex^ in different pHs and buffers, with resolution ranging from 2.5 to 3.5 Å (Supplementary Figures S6–S8). Based on our biochemical results, we performed cryo-EM data collection of NendoU^hex^ in buffers where the enzyme showed maximum activity and reduced activity, such as PBS at pH 6.0, Bis-Tris at pH 6.0 (two more active conditions) and HEPES at pH 7.5 (a less active condition), detailed in Supplementary Table S1. Despite their similarities, models showed consistent differences in the switch region (Figure 3). For samples in HEPES at pH 7.5, we observed that the canonical form of interacting switch regions assumes a more closed and mobile conformation in comparison with known crystal structures (Figure 3A). However, in cryo-EM models obtained in PBS at pH 6.0 and Bis-Tris at pH 6.0, we saw a less mobile and more open conformation of switch regions, much more like known crystal structures (Figure 3B). The overall structure of the hexamer is also deeply affected by these distinct conformations of the switch region, where in pH 7.5 we observe a more contracted form of the barrel (Figure 3A). The 3DVA Supplementary movie #1 shows details of the contracting front and top view of the closed form of NendoU. In contrast, in pH 6.0 conditions (where the enzyme is more active), we see that the structure is more rigidly defined in the open conformation, as can be seen in Supplementary movie #2 and in Figure 3B.

In the NendoU dsRNA complex solved by Frazier *et al.* (59), the positively charged switch region can be found in the open form and seen to form key interactions with the nucleic acid, indicating that this region is involved in positioning the RNA into the active site in a form of the enzyme that resembles the open state (Figure 2D). In fact, this open–closed form model seems to agree with our biochemical data showing that the enzyme is far more active in acidic than in basic pH, where the switch seems to be found in the closed conformation. In the dsRNA complex, we can also see that the MD active site region of the adjacent auxiliary NSP15 chain is twisted relative to the open and closed forms of NendoU (Figure 3C), while the occupied active site from the productive (bound to RNA in the active site) chain is less altered among the states (Figure 3C). In the movie generated by 3DVA of samples in HEPES at pH 7.5 (Supplementary movie #1) and PBS at pH 6.0 (Supplementary movie #2), we can see the wobbling movement of these chains relative to each other and it highlights the conformational changes of the switch region, similar to the movements observed by Pilon *et al.* (11). However, while those authors proposed that the wobbling movement was the induced fit of the enzyme processing mechanism (11), our data indicate that this movement is the shift between the open and closed states of the enzyme, which regulate the enzyme active–inactive states, while the induced fit will occur with the conformational shifts of MD and switch regions within the constraints of the open state.

This might also explain why the structure of Frazier *et al.* (59) contains only one strand of nucleic acid at any time, even though all other active sites are unoccupied during the binding, again agreeing with our biochemical characterization. This flexible mode of action involving two adjacent NSP15 chains interconnected by the switch region might explain not only why the cooperative index of NendoU is two (and not one, three or six), but also how and why pH affects the enzyme activity so drastically (Supplementary Table S1). That model also diverges from recently proposed models in which bottom and top trimers would act independently in substrate recognition and processing (11).

### NendoU polar surface allows stacking in the shape of filaments

**Figure 4.**
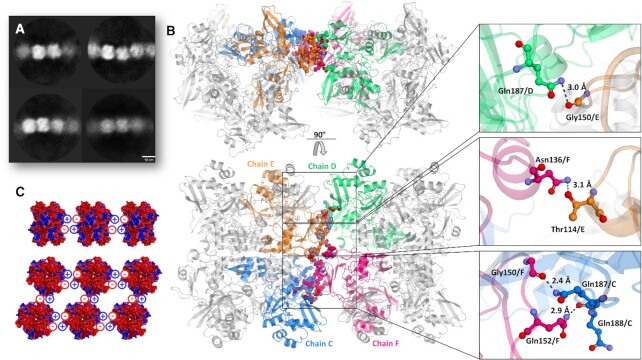
Overview of supramolecular organization of NendoU. (**A**) 2D classification of selected filaments of NendoU observed from samples collected in Bis-Tris at pH 6.0. (**B**) Crystal structure of NendoU in the presence of oligo(dT) reveals high resolution details of contacts between the switch region of two NendoU hexamers (PDB 7KF4). The protein is depicted as a cartoon, with chains C, D, E and F colored in blue, green, orange and pink, respectively. Contacting residues are shown as colored sticks. (**C**) Stacking model based on the surface charge of NendoU. The structure of NendoU is colored according to its electrostatic potential projected on surface charge (–0.5 to 0.5 f kJ/mol/e in a red-white-blue color model).

A general observation we made was that particles from samples in basic pHs, such as HEPES at pH 7.5, tend to be individual particles (Supplementary Figure S9), while particles from samples obtained in more acid pHs, such as Bis-Tris at pH 6.0 and PBS at pH 6.0, tend to be arranged side by side, forming filaments (Supplementary Figure S10A), which is a common mechanism of allosteric control of enzyme activity (61). By using a large box size (512 × 512 px) for the sample in Bis-Tris at pH 6.0, we were able to even classify and generate a low-resolution initial model of this dodecamer particle stack (Supplementary Figure S10B, C), which had also been observed in native mass spectroscopy (Supplementary Figure S4). Using helical processing tools, we have been able to generate 2D classes of hand selected filaments, and data consistently indicate that they are composed of C1 symmetrically paired units of the hexamer (Figure 4A). The positioning of particles also indicates that hexamers are paired by the switch regions, but resolution of the model obtained by cryo-EM was not sufficient for describing these interactions (Supplementary Figure S10C). However, the most drastic similar effect was obtained when we added the DNA oligo(dT)_30_ to the samples in Bis-Tris at pH 6.0 and in PBS at pH 6.0, where we saw the immediate formation of 2D crystals of NendoU in the frozen grid, which somehow had induced the same pattern of arrangement of particle in all directions (Supplementary Figure S11). Due to that, we tried to use this condition to obtain a crystal structure of NendoU.

Typical crystals of NendoU are usually obtained in a hexagonal space group, with a dihedral asymmetric unit that can be symmetry expanded to generate the full hexamer. In these, the crystal packing resembles a chess board arrangement of biological units, as for example in PDBs 7KEG and 7KEH, with solvent content of ∼74% (Supplementary Figure S12). However, when NendoU^mon^ was incubated with oligo(dT), we observed that the generated crystals were obtained in an orthorombic space group with 55% solvent content and biological units packed side by side as observed in the cryo-EM oligo(dT) samples, as exemplified here in PDB 7KF4 (Supplementary Figure S12). Furthermore, the same crystal pattern was also observed for NendoU^mon^ and NendoU^hex^ when incubated with other DNA sequences such as primers (data not shown), suggesting that this packing is somehow induced by the presence of DNA. If we compare the arrangement of adjacent hexamer units in the crystal structure (Figure 4B) with the low-resolution processing of cryo-EM filaments (Figure 4A; Supplementary Figure S10), it can be inferred that they follow the same pattern, with hexamers positioned side by side in a C1 arrangement.

The high resolution of the crystal structure (PDB 7KF4) allowed us to depict the contacts taking part in the filament pattern, which involve the open form of the switch region of two adjacent hexamers, including the hydrogen bounds between Gln187 and Gly150, Asn136 and Thr114, Gly150 and Gnl187, and Gln152 and Gln188 (Figure 4B). Furthermore, polar surface structural analysis showed that this pattern allows the perfect battery-like piling of the negatively charged S cavity of one hexamer into the positively charged switch region of the adjacent hexamer (Figures 2 and 4C), indicating that the biological function of these two regions is related to the formation of large order supramolecular agglomerates. Further, we can see that this structure is in closed form and resembles more 7ME0 (r.m.s.d of 0.36 Å for 347 residues) than 7RB0 (r.m.s.d of 1.31 Å for 347 residues), indicating that the stacking traps protein in the inactive conformation. To investigate the biological role of this still undescribed behavior, we generate the mutant T114L to avoid the formation of the hydrogen bound between Asn136 and Thr114 (Figure 4B). Indeed, this mutant showed much higher activity at higher concentrations of RNA substrate than the wild type (Figure 1F), suggesting that interactions between adjacent NendoU particles can down-regulate enzymatic activity by occluding the RNA-binding region. This is in line with a recent proposed model showing that regulation of SARS-CoV-2 replication and transcription could be driven by a negative feedback model where NendoU regulates the balance between genomic and subgenomic RNAs (62, 63). However, more data are required to validate this mechanism and the association with the allosteric mechanism demonstrated here. Notwithstanding, the ability of NendoU to stack and occlude its active site could explain how certain sequences might be preferably degraded by NendoU, even in the absence of a specific active site. Yet, given the complexity of the NendoU biochemical profile, we have not been able to fully understand the role of the switch region in enzyme regulation to date. More *in situ* studies are required to confirm the existence of these molecular entities inside infected cells. It is important to state that despite our efforts, we have not been able to identify any electron density suggesting a complex with nucleic acids.

### Crystallographic fragment screening of SARS-CoV-2 NendoU

To identify starting points for new therapeutics, during the initial months of COVID-19, the XCHEM team and international collaborators performed large crystallographic fragment screens against multiple SARS-CoV-2 proteins, including the viral Main protease, the Nsp3 macrodomain and the helicase Nsp13 (64–66), which led to the rapid development of new potential antivirals (67). Here we report the results obtained during the fragment screening campaign against NSP15 NendoU.

**Figure 5.**
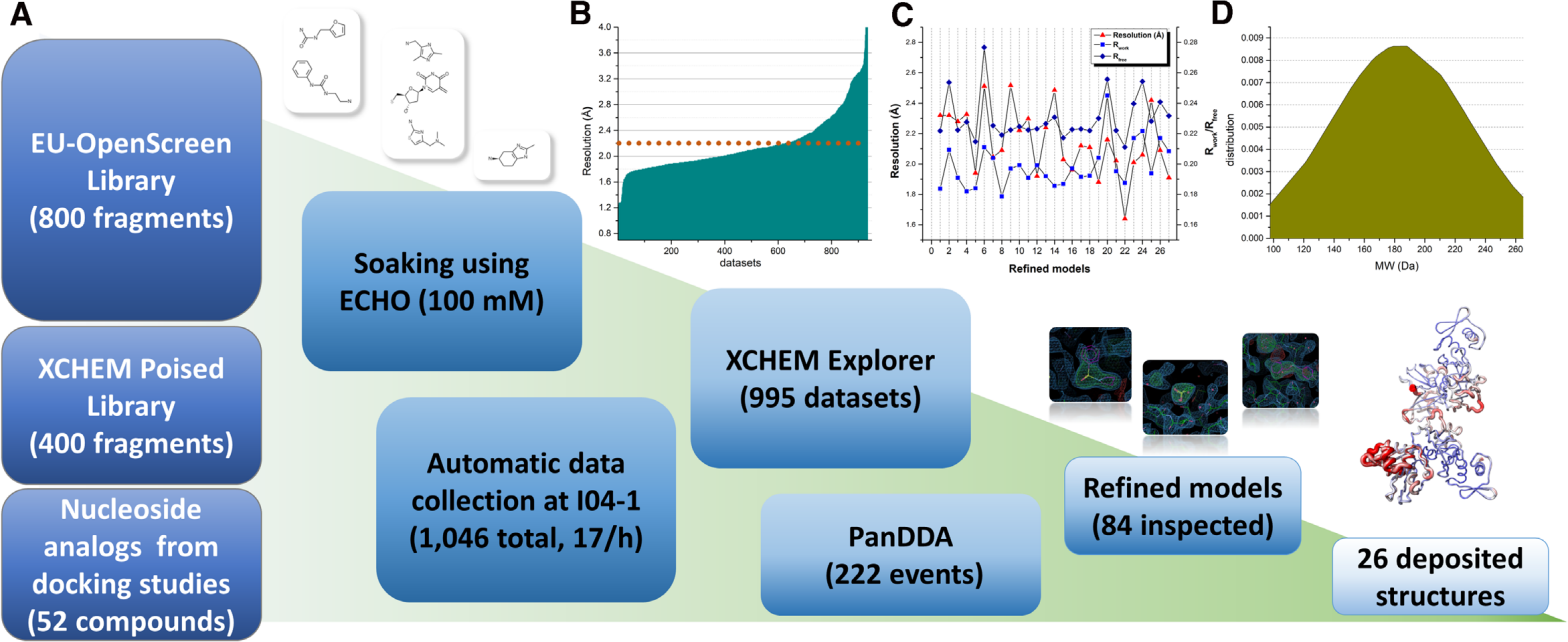
Overall schematic showing steps and data processing of the fragment screening campaign against NendoU. (**A**) Schematic summarizing steps from library screening to refinement and deposition of models. Panels show event maps of selected fragments (1.0 sigma) and the overlap of all structures colored according to B-factors. (**B**) Graph showing the obtained resolution per dataset collected and processed. The dotted line marks the average resolution of all 997 usable datasets (2.2 Å). (**C**) Graph showing resolution and refinement statistics of the final models deposited. Resolution is colored in red triangles, R_work_ is colored in blue squares and R_free_ is colored in dark blue diamonds. (**D**) Normal distribution graph of the molecular weitght from identified fragments.

For the fragment screening campaign, the chosen crystal system was obtained in a citrate condition and hexagonal space group *P*6_3_, containing two NSP15 monomers in the asymmetric unit arranged in a dihedral symmetry, which can be symmetrically expanded to reveal the full hexameric form of NendoU inside the crystal lattice. Three libraries of compounds were used for soaking, i.e. the XCHEM Poised Library (42), the OPEN-EU DRIVE fragment library and a set of 52 nucleoside analogs (Figure 5). Over 1200 crystals were soaked with different fragment compounds or nucleoside analogs, resulting in 997 datasets collected and processed, with an average resolution of 2.2 Å (Figure 5B). Despite the high concentration of ligands used for soaking (100 mM), as well as the large amount and high quality of crystallographic data, only 26 crystal complexes of fragments or nucleosides (2.5%) with NendoU were obtained, highlighting the difficulties in identifying fragments for this crystal system, probably related to the large solvent content in the crystal unit cell (74%). From the 26 ligand complexes elucidated, we found that 13 ligands were found bound to the two chains, while the other 13 were only identified in one of the chains, which can be explained by the distinct dynamics between top and bottom trimers, related to the mechanism of action of NendoU (11). The average molecular weight of fragments identified was 183 ± 43 Da. The summarized information on PDB accession codes for each complex, resolution, refinement and processing statistics, ligand structure and chain location are available in Supplementary Table S1.

**Figure 6.**
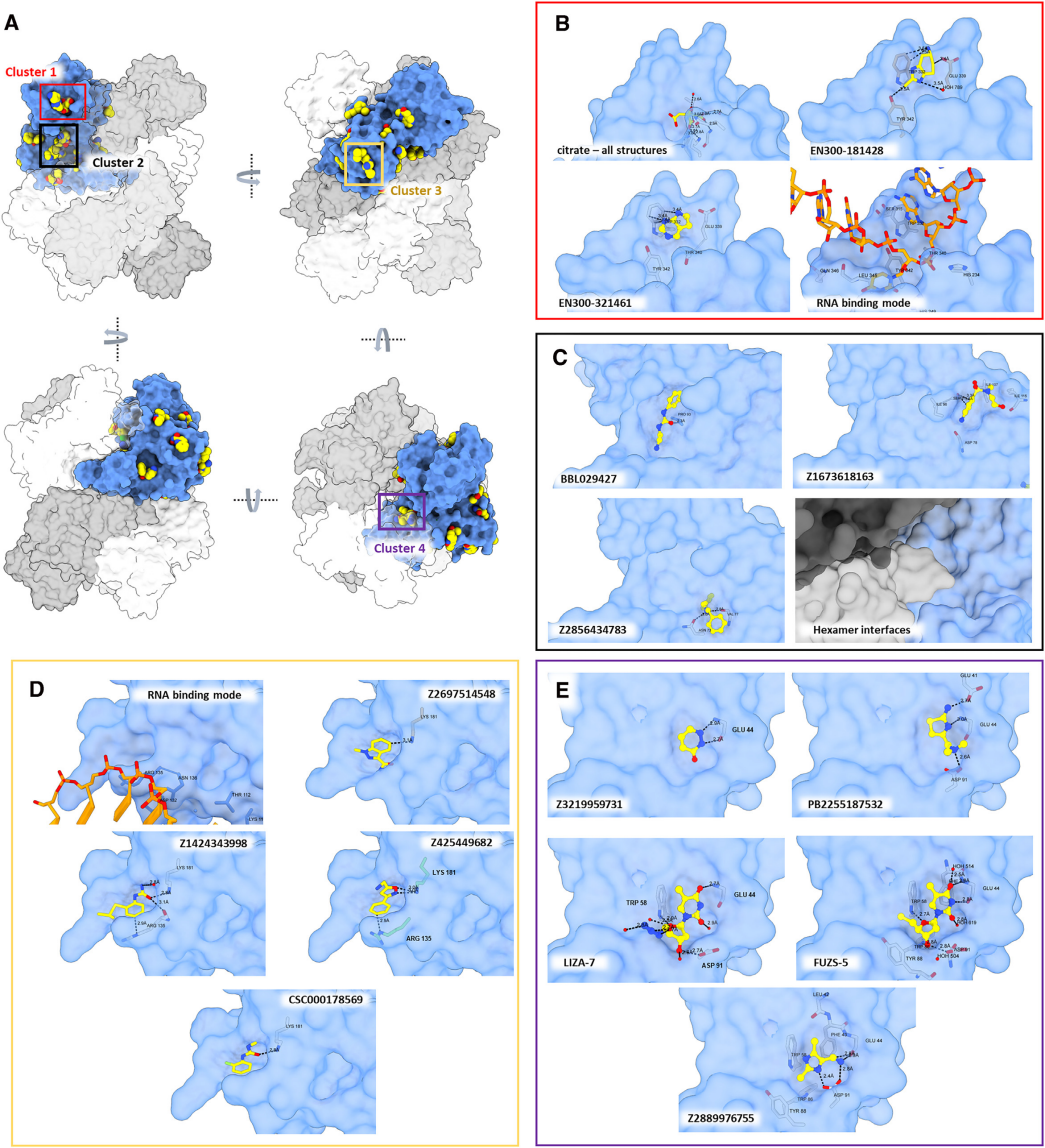
Overall visualization of fragments bound to NendoU. (**A**) Overall view of all NendoU sites bound to fragments in multiple orientations. All chains containing fragments were supperposed into one, colored as blue surface, while adjacent chains of biological units are colored in shades of gray. Colored squares highlight the cluster of fragments identified. (**B**) Detailed view of cluster 1 (active site) showing citrate, multiple fragment contacts and the RNA binding mode. (**C**) Detailed view of cluster 2 showing multiple fragment contacts and the oligomerization interface. (**D**) Detailed view of cluster 3, showing multiple fragment contacts and the RNA binding mode. (**E**) Detailed view of cluster 4, showing multiple fragment contacts with NendoU. In all, the NendoU surface is colored in blue, fragments are depicted as sticks or spheres with yellow carbons, while interacting NendoU residues are depicted as gray carbon sticks. RNA carbons are colored in orange, and coordinates were obtained from PDB 7TJ2.

To simplify our analyses, we superposed chains A and B of each complex onto chain A of a hexamer structure (PDB 7KF4), so that we can map all the identified druggable cavities in the context of the hexameric complex (Figure 6A). Also, we used the recent structure of NendoU in complex with dsRNA (PDB 7TJ2) to inspect any of these sites that are in the path of the RNA binding surface (59). We found 14 distinct binding sites spread across the NendoU surface, one at the ND, nine at the MD and four at the CD. In the hexamer context, these sites are distributed across all the external and internal surfaces of the complex (Figure 6A). Among those, we identified four sites where two or more ligands have formed clusters, which opens up the possibility of fragment expansion.

Unfortunately, the NendoU active site, which would be a primary target for the development of a competitive inhibitor, was occupied by a citrate molecule that originated from the crystallization condition in all refined datasets (Figure 6B), which resulted in a small number of binders to this site. The only identified fragments in this site were two aromatic fragments, EN300-181428 and EN300-321461, forming a π–π stacking interaction with Trp332 (Figure 6B). This is consistent with the mode of action of NendoU, which prefers an aromatic purine at the +2 position together with the intrinsic requirement for a uridine in the cleavage position.

The second cluster (cluster 2) mapped is in a conserved region of the MD, which is a critical region of the oligomerization interface (Figure 6C). Here we identified a large cavity in which fragments BBL029427, Z1673618163 and Z2856434783 are forming hydrogen bonds with multiple MD residues, including Pro93, Ser97, Asn73 and Val77 (Figure 6C). These three fragments are spread across the cavity, permitting a good exploration of its chemical space and potential for fragment linking or merging.

Another possible allosteric site for the development of new inhibitors is that identified in the third cluster (cluster 3), which is also a conserved region located at the bottom of the MD, where we found a cavity containing fragments Z2697514548, Z1424343998, Z425449682 and CSC000178569, forming different contacts with residues Arg135 and Lys191 (Figure 6D). The structure of NendoU in complex with dsRNA reveals that the positive charges of Arg135 and Lys191 side chains are key for positioning long nucleic acids into the active sites of NendoU (59).

**Figure 7.**
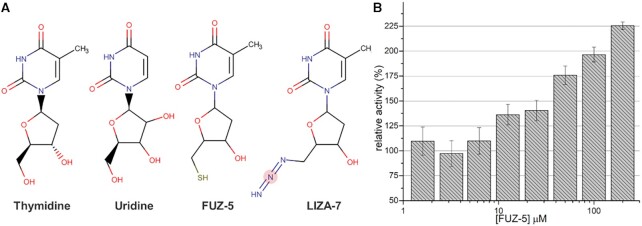
Fragments bound to cluster 4 of NendoU. (**A**) Structural comparison between thymidine, uridine, FÜZS-5 and LIZA-7 fragments. (**B**) Effect of different FÜZS-5 concentrations on NendoU relative enzymatic activity determined using a fluorogenic substrate.

The most curious site identified in our experiments was cluster 4, which is a conserved site located in the interfaces of the ND and MD with the NSP15 domain of the neighboring monomer, in a cavity located inside the barrel shape of NendoU (Figure 6E). In addition to the aromatic fragments Z3219959731, PB2255187532 and Z2889976755, we also identified two nucleoside analogs tightly bound to this allosteric site (Figure 6E). The two thymidine analogs are 5′-azido-5′-deoxy-thymidine (LIZA-7) and 5′-deoxy-5′-thiothymidine (FÜZS-5), with their deoxyribose unit showing two productive hydrogen bonds with Trp58 and Asp91, as well as a π–π stacking between the nucleoside base and Trp58. To further investigate the role of this allosteric site, we have examined the effect of FÜZS-5 on the stability and enzymatic activity of NSP15, with a thermal stability assay and a fluorescence-based enzyme activity assay, respectively. Notably, FÜZS-5 increased the thermal stability of NendoU by 0.4°C, suggesting a stabilizing role for the compound itself, as well as other potential binders of this allosteric site. Furthermore, the enzyme activity assay revealed FÜZS-5 to increase the reaction rate, in a closely linear, concentration-dependent fashion (Figure 7). Together with the X-ray structure, these results suggest the positive allosteric modulation of NendoU by FÜZS-5, indicating a previously unknown, positive feedback mechanism for the activity of NendoU. The close structural analogy of FÜZS-5 to the natural nucleoside thymidine (as well as uridine) suggests that this positive modulatory mechanism might be triggered under physiological conditions as well, by endogenous nucleosides. More studies are being conducted to clarify this mechanism.

Besides the four identified clusters, we were also able to map several different druggable cavities spread across the hexamer structure, including fragments EN300-1605072, Z2443429438, Z425449682, Z59181945, Z239136710, Z1530301542, Z18197050, EN300-100112, Z319891284, Z68299550, Z56900771 and Z31504642, which are all depicted in Supplementary Figure S14. In summary, our data allowed us to map several new sites for the development of allosteric inhibitors that had never been described for NendoU, including multiples sites that might be used to block oligomerization or RNA recognition (Figure 6A). The ongoing expansion of selected fragments and characterization will be critical for validating the therapeutic potential of these sites, opening up opportunities for the development of new allosteric inhibitors of NendoU.

### Fragments caused the disruption of the crystal structure of the NendoU active site

During our analysis of fragment screening data, we identified a set of datasets that had a completely disrupted chain A active site, causing an extreme increase in the B-factor of this region (Supplementary Figure S15). A detailed inspection of these datasets showed no significant twinning or other crystal pathology that would explain such poor electron density. Yet, despite our efforts, we were not able to obtain a refined map of this region, or identify fragments bound to it. Still, given all the previous observations related to mobility of one individual active site and the lack of fragments bound to the active site, it is likely that these fragments are somehow interacting with the active site region and causing the disruption of the processive or auxiliary chains only. These are fragments Z1741794237, Z1713595338, Z85934875, Z1437171658, Z1636723439, Z2856434903, Z1262327505, Z1269638430, Z927400026 and POB0120, and their structures are depicted in Supplementary Figure S15B. Based on our biochemical model of active–inactive forms, it is possible that these fragments are causing a shift between the active and inactive state. Further biochemical assays will be critical to understand the effect of these fragments on the enzyme activity. Although we could not elucidate these structures, the datasets of these fragments can be found in the Supplementary datasets file. More studies are required to understand the biological impact of these fragments on NendoU activity and define their mode of interaction.

## DATA AVAILABILITY

Any other data can be supplied upon reasonable request. Crystallographic coordinates and structure factors for all structures have been deposited in the PDB with the following accession codes 5S6X, 5S6Y, 5S6Z, 5S70, 5S71, 5S72, 5SA4, 5SA5, 5SA6, 5SA7, 5SA8, 5SA9, 5SAA, 5SAB, 5SAC, 5SAD, 5SAE, 5SAF, 5SAG, 5SAH, 5SAI, 5SBF, 7N7R, 7N7U, 7N7W, 7N7Y, 7N83, 7KEG, 7KF4, 7KEH, 7RB0, 7RB2 and 7ME0. EMD data are available with the following accession codes EMD-24391, EMD-24392 and EMD-23786.

## Supplementary Material

gkad314_Supplemental_Files
