# Supplementary material for: Allosteric regulation and crystallographic fragment screening of SARS-CoV-2 NSP15 endoribonuclease
Source: Nucleic Acids Res. 2023 Apr 28;51(10):5255–70. doi: 10.1093/nar/gkad314 (PMC10250223; doi:10.1093/nar/gkad314)

**Supplementary Material Information**

**Fig. S1.** A) Diagrams of different NendoU constructs. B) Gel filtration profile of NendoU^hex^, showing peaks of hexamers (1), trimers (2) and monomers (3) observed during protein production. C) Gel filtration profile of NendoU^mon^, showing peaks of monomers (4) observed during protein production. Peak 1 contained the pulled fraction used for preparing NendoU^hex^ sample. Peak 4 contained the pulled fraction used for preparing NendoU^mon^ sample.


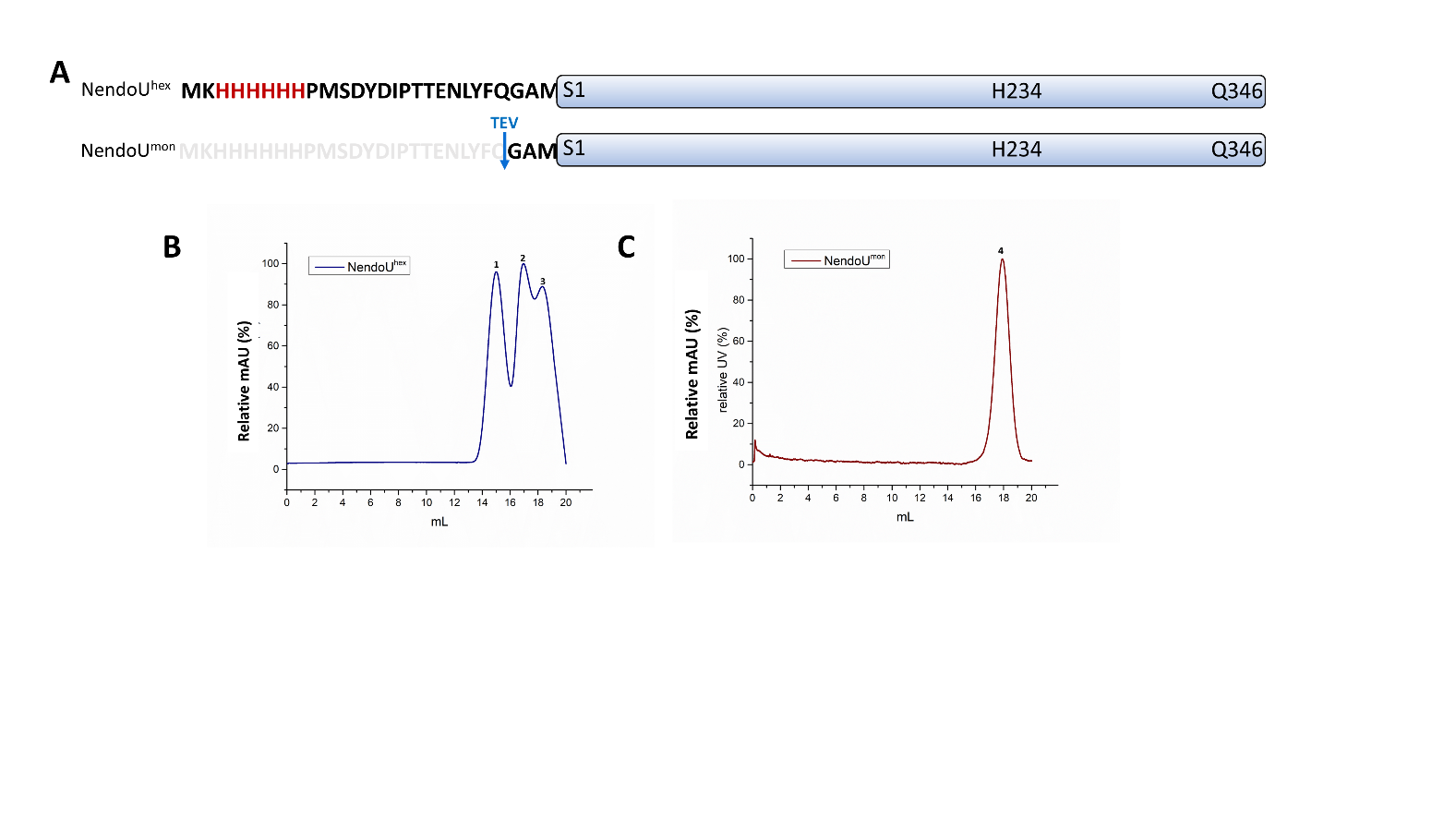


**Fig. S2.** Analytical Size exclusion chromatography profile of NendoU oligomeric forms. A) calibration curve using standard proteins. B) Size exclusion chromatography profile of purified NendoU^hex^, with a total mass of 204 kDa. C) Size exclusion chromatography profile of purified NendoU^mon^, with a total mass of 34 kDa. In B and C, the sample containing peak is pointed with a black arrow.


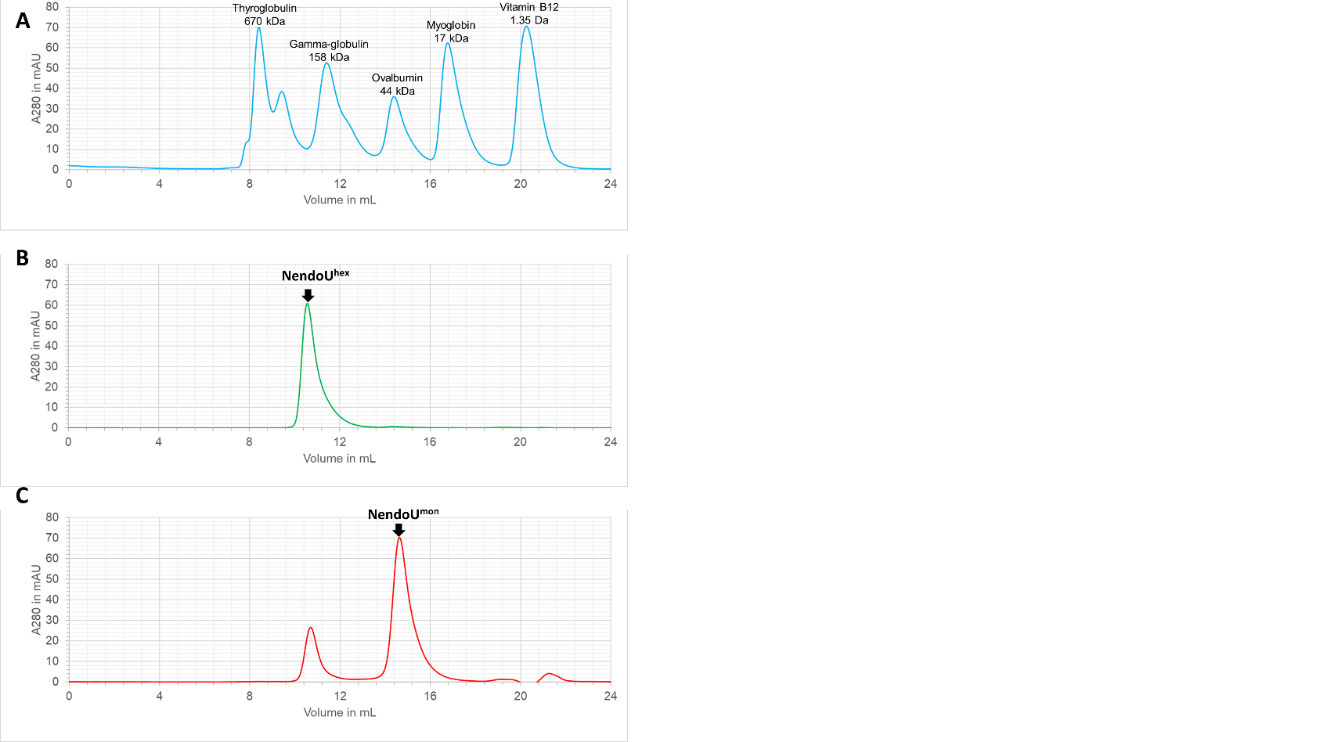


**Fig S3.** Mass spectroscopy profile of NendoU. In A), the mass spectra of full cleaved NendoU^mon^. In B), the mass spectra of NendoU^hex^ showing the mass of NSP15 with the additional expression N-terminal residues.


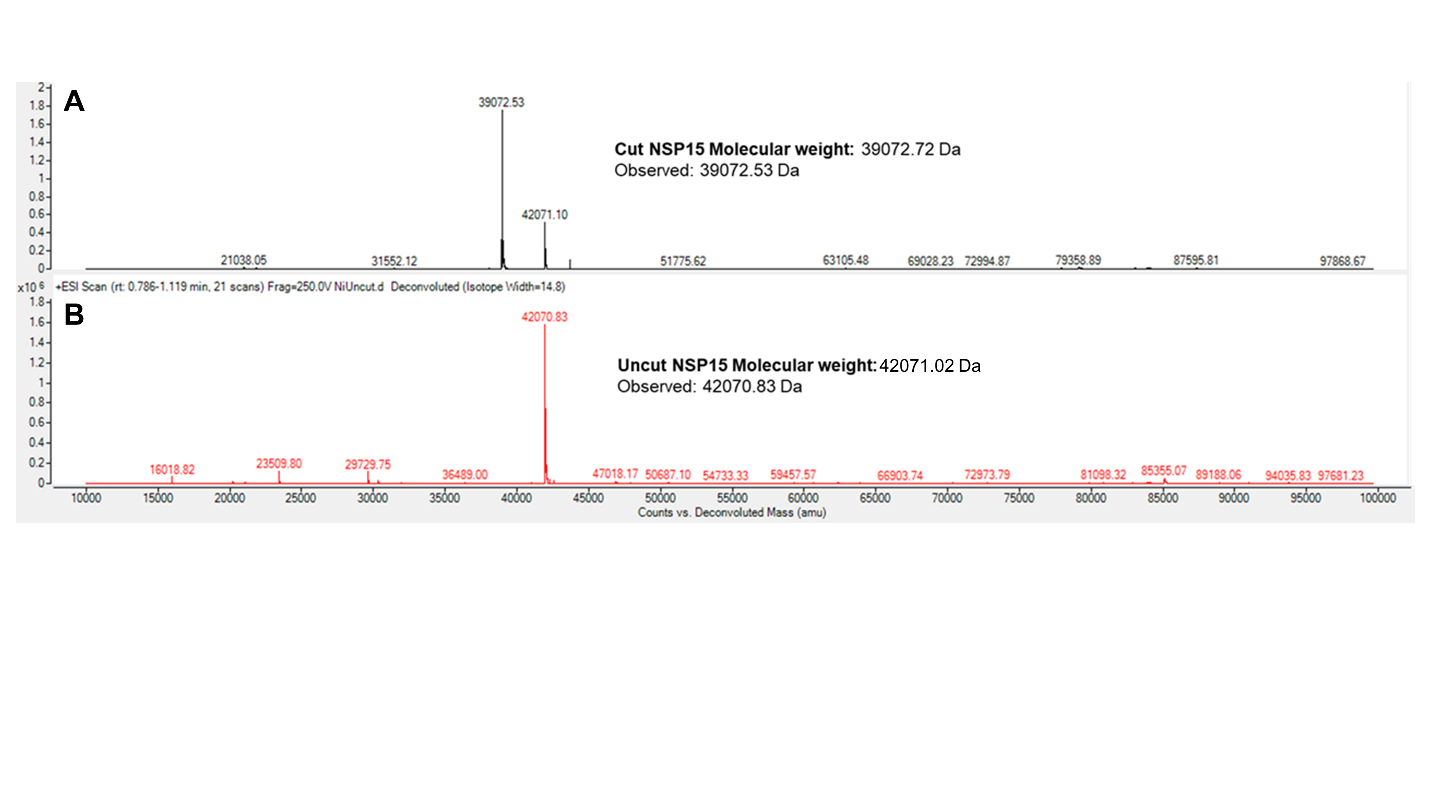


**Fig S4.** Native mass profiles of NendoU^mon^ and NendoU^hex^. A) Native mass spectra of NendoU^mon^ sample, showing that most of the sample is presented as folded monomers. Is also possible to observe the presence of folded dimers, trimers and pentamers/hexamers B) Native mass spectra of NendoU^hex^ sample, showing that the majority of the sample is presented as folded hexamers or folded monomers. Is also possible to observe the presence of folded dodecamers in the sample.


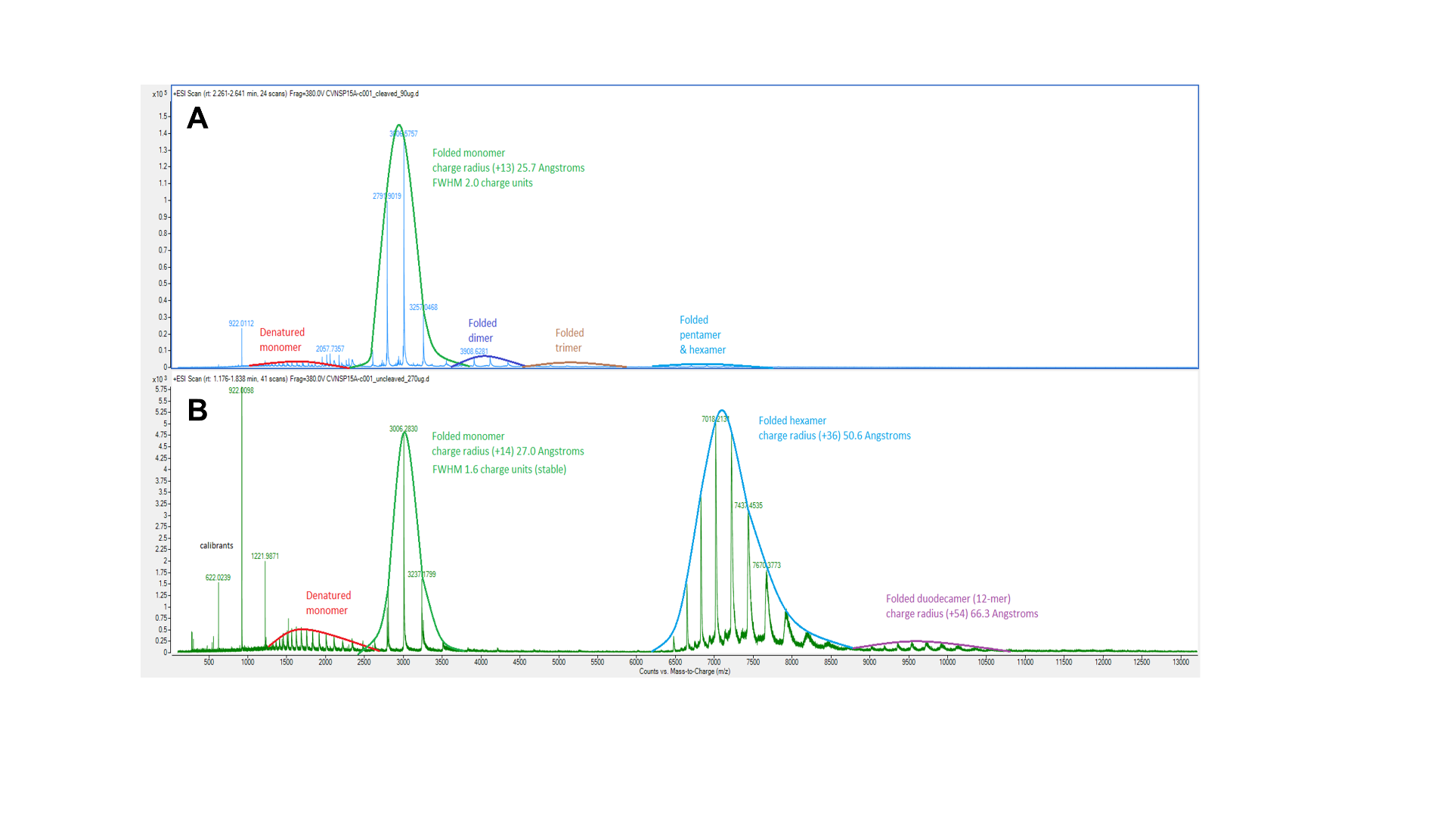


Fig S5. A) Crystal structure of the three domain NSP15 monomer. N-terminal domain is colored in shades of blue, middle domain is colored in shades of green and C-terminal catalytic domain is colored in shades of red yellow. B) Front (left) and top (right) view of the NendoU hexamer. One of the chains is showed as cartoon (colored as A), while the other five are showed as spheres. C) Topology model of NSP15, following the same color pattern of A. Cylinders represent helices, with arrows are representing strands.


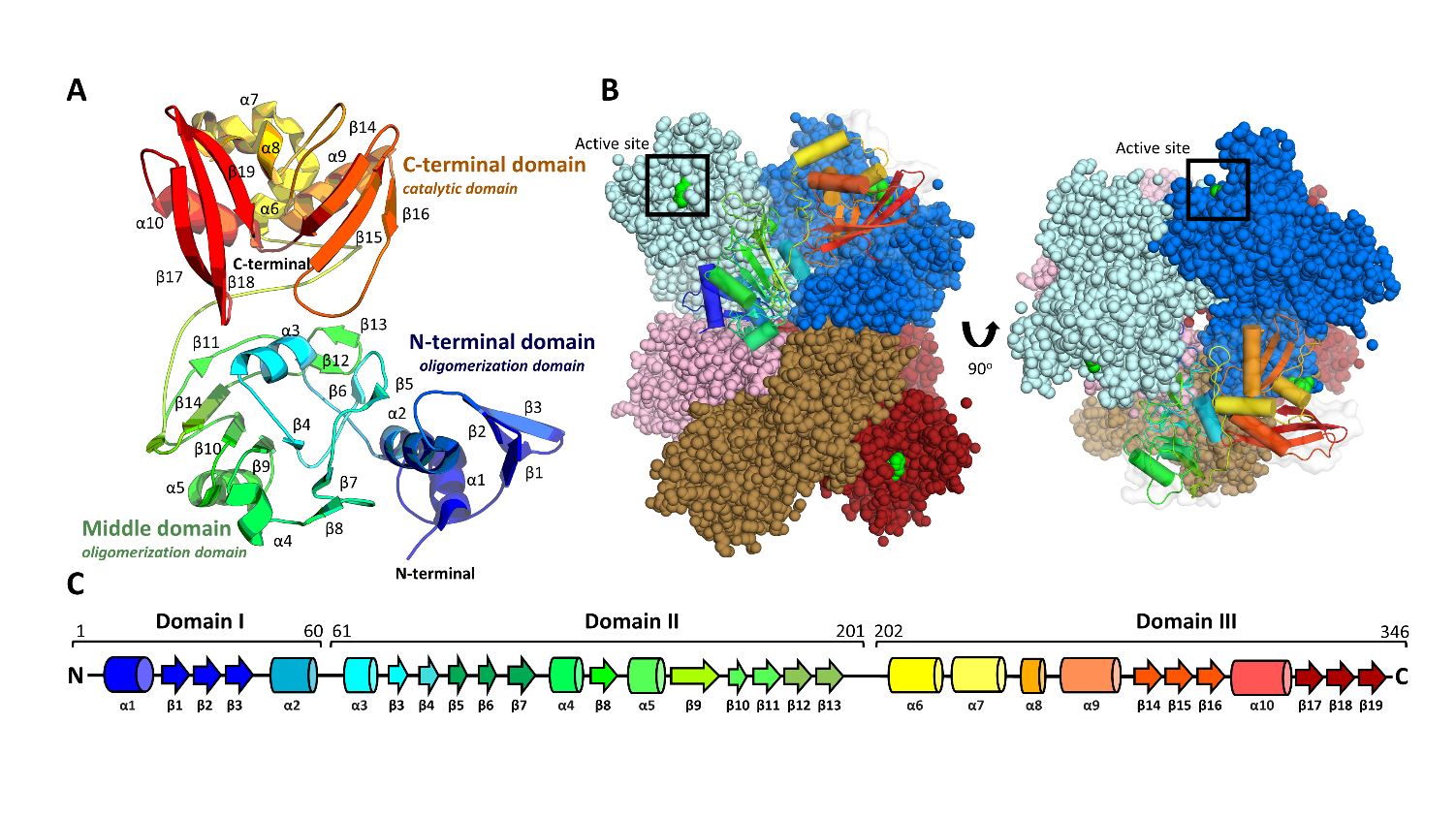


Fig. S6. Cryo-EM data processing schematic of NendoU^hex^ collected in HEPES pH 7.5.


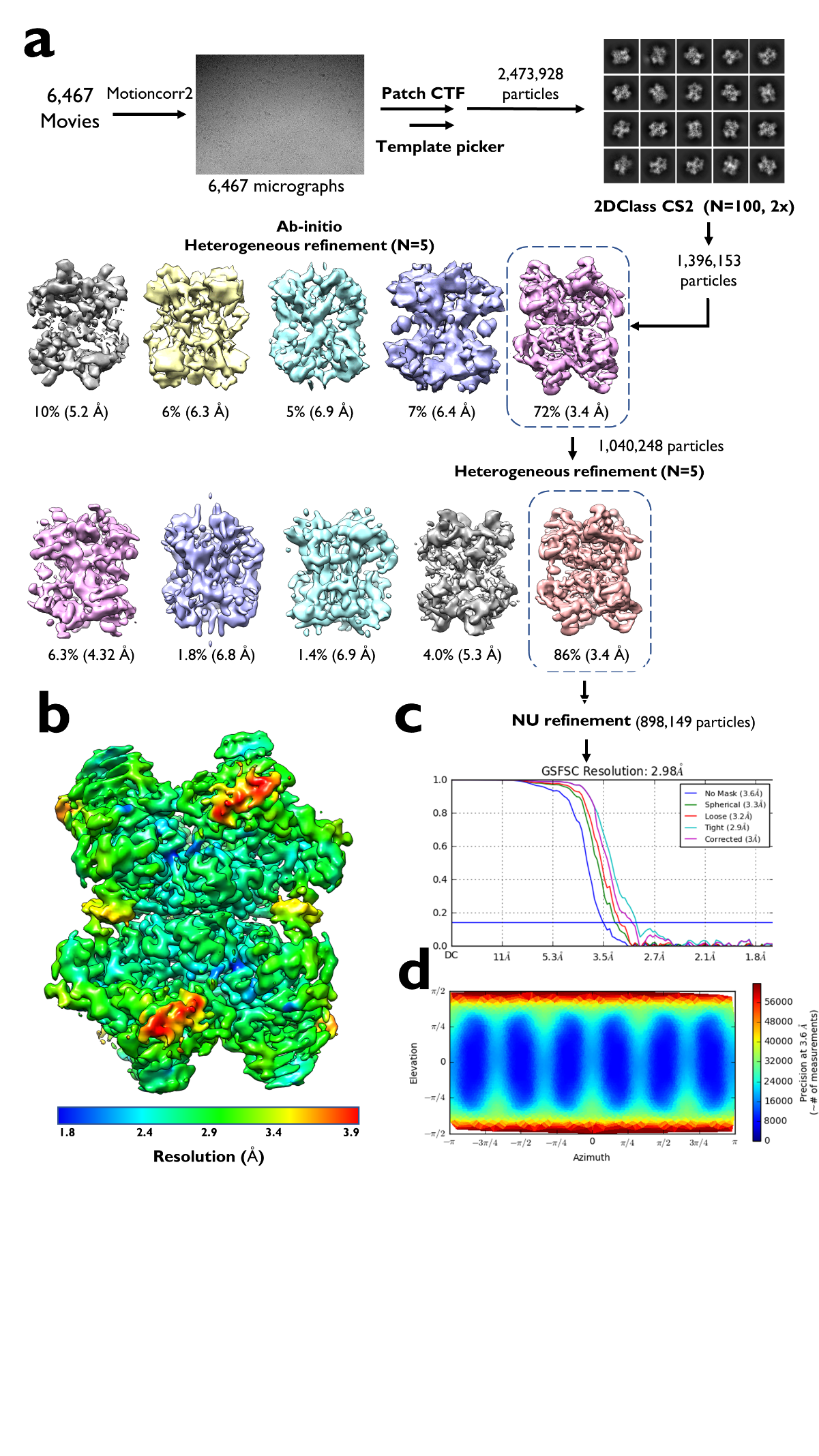


Fig. S7. Cryo-EM data processing schematic of NendoU^hex^ collected in BIS-Tris pH 6.0.


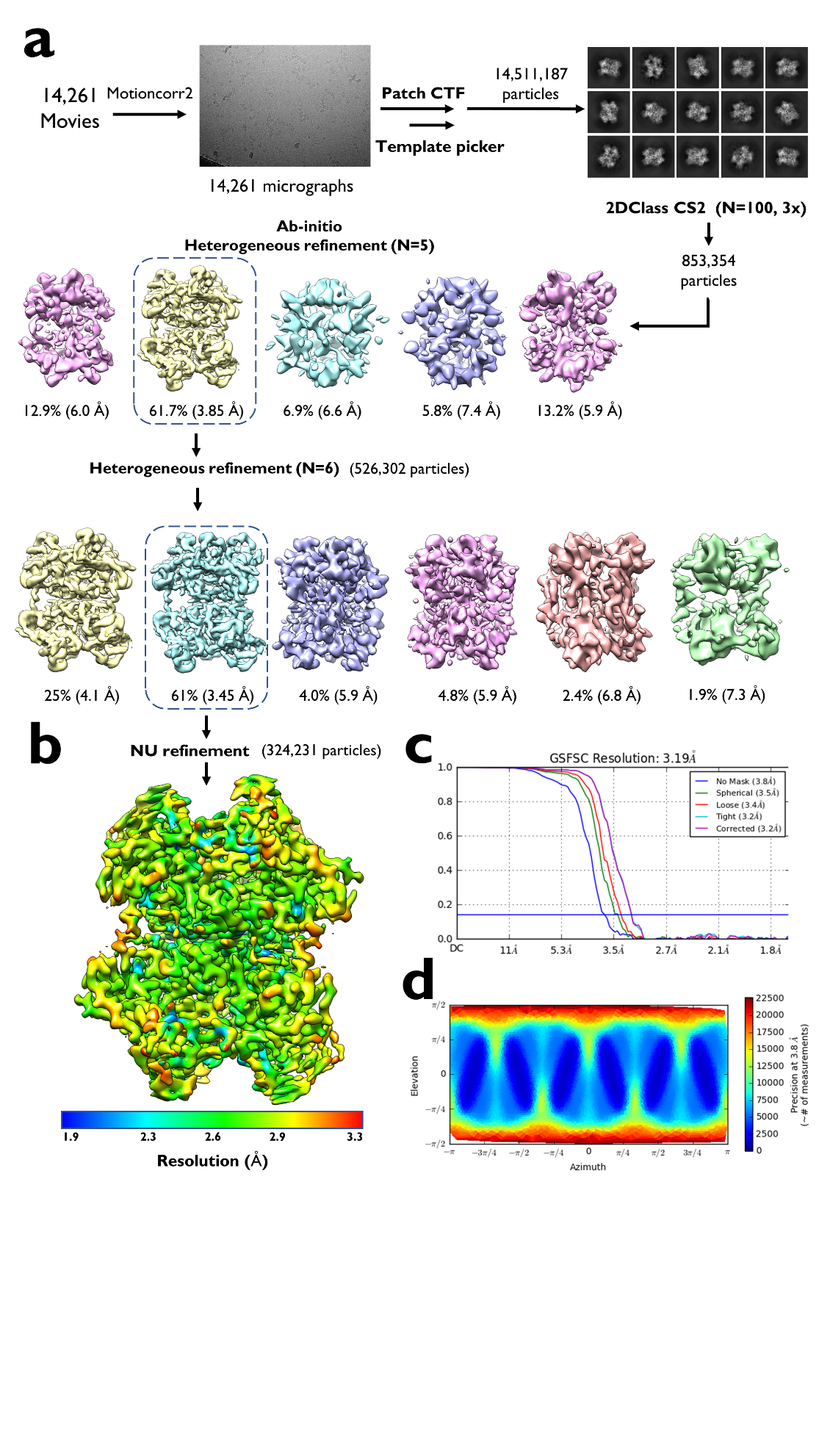


Fig. S8. Cryo-EM data processing schematic of NendoU^hex^ collected in PBS pH 6.0.


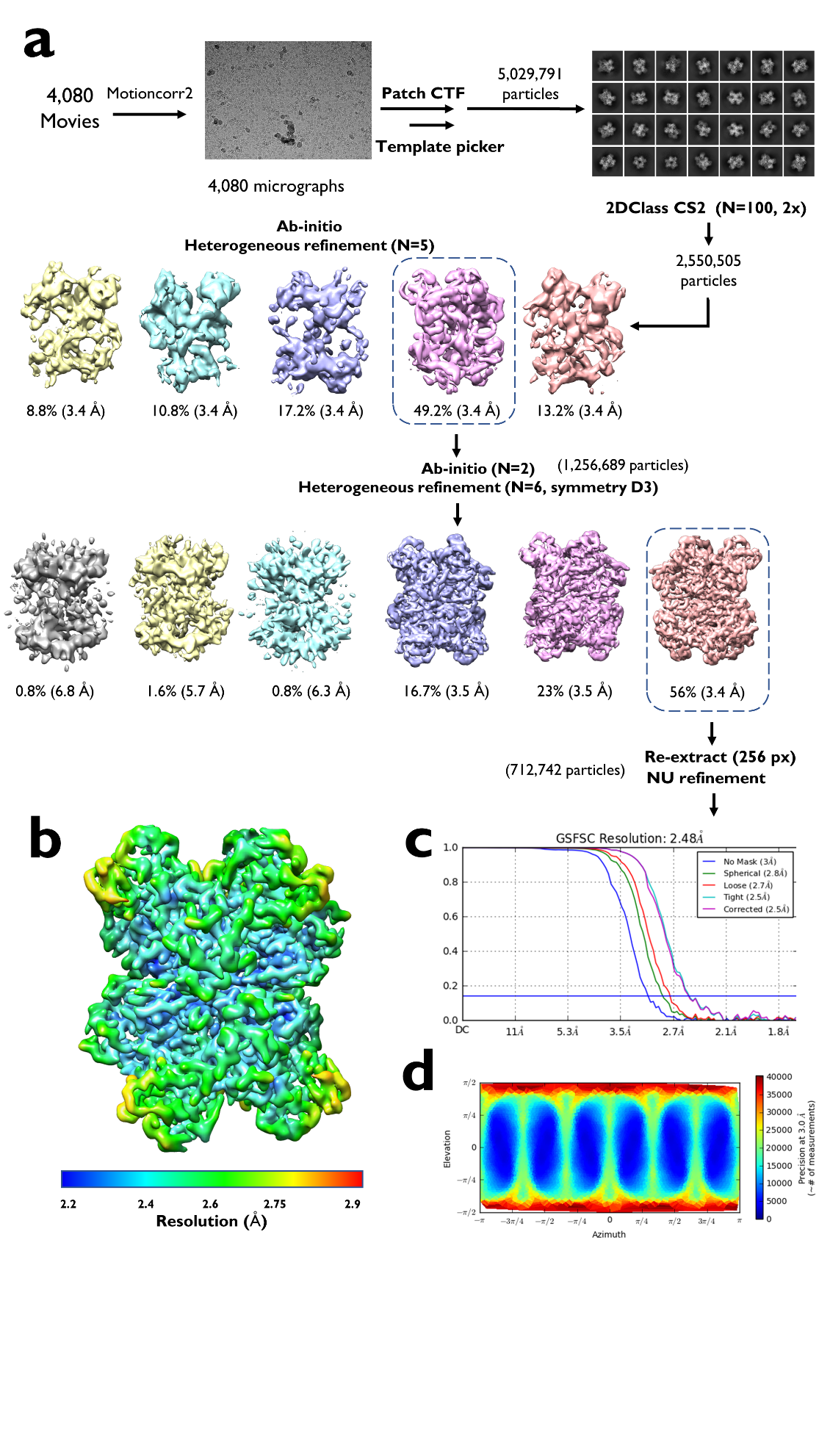


Fig. S9. A and B) Cryo-EM images of NendoU^hex^ collected in HEPES pH 7.5.


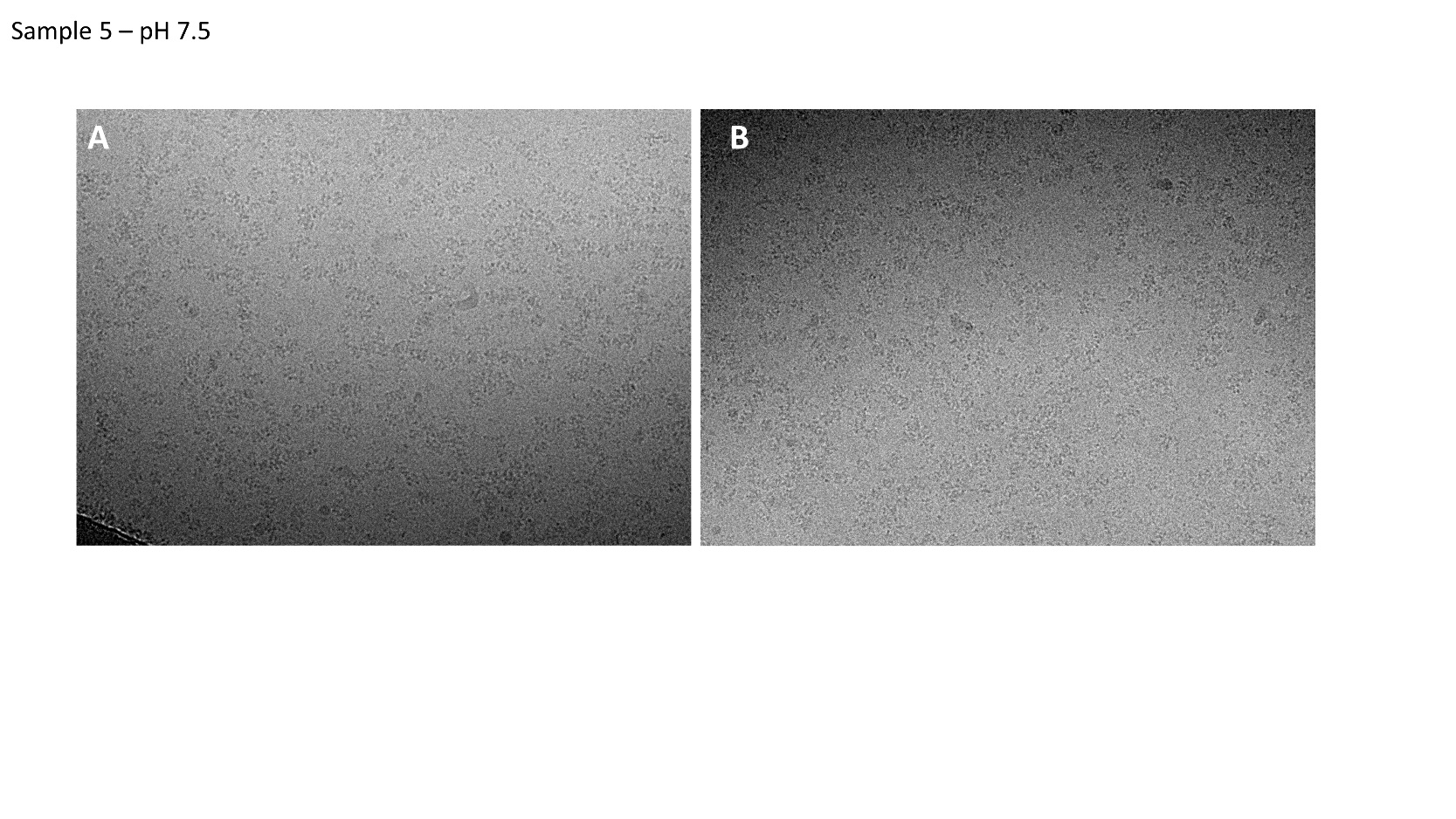


Fig. S10. A) Cryo-EM images of NendoU^hex^ collected in Bis-tris pH 6.0. B) 2D classes of stacked particles. C) Low resolution volume reconstruction of stacked particles, with fitted model from PDB 7KF4.


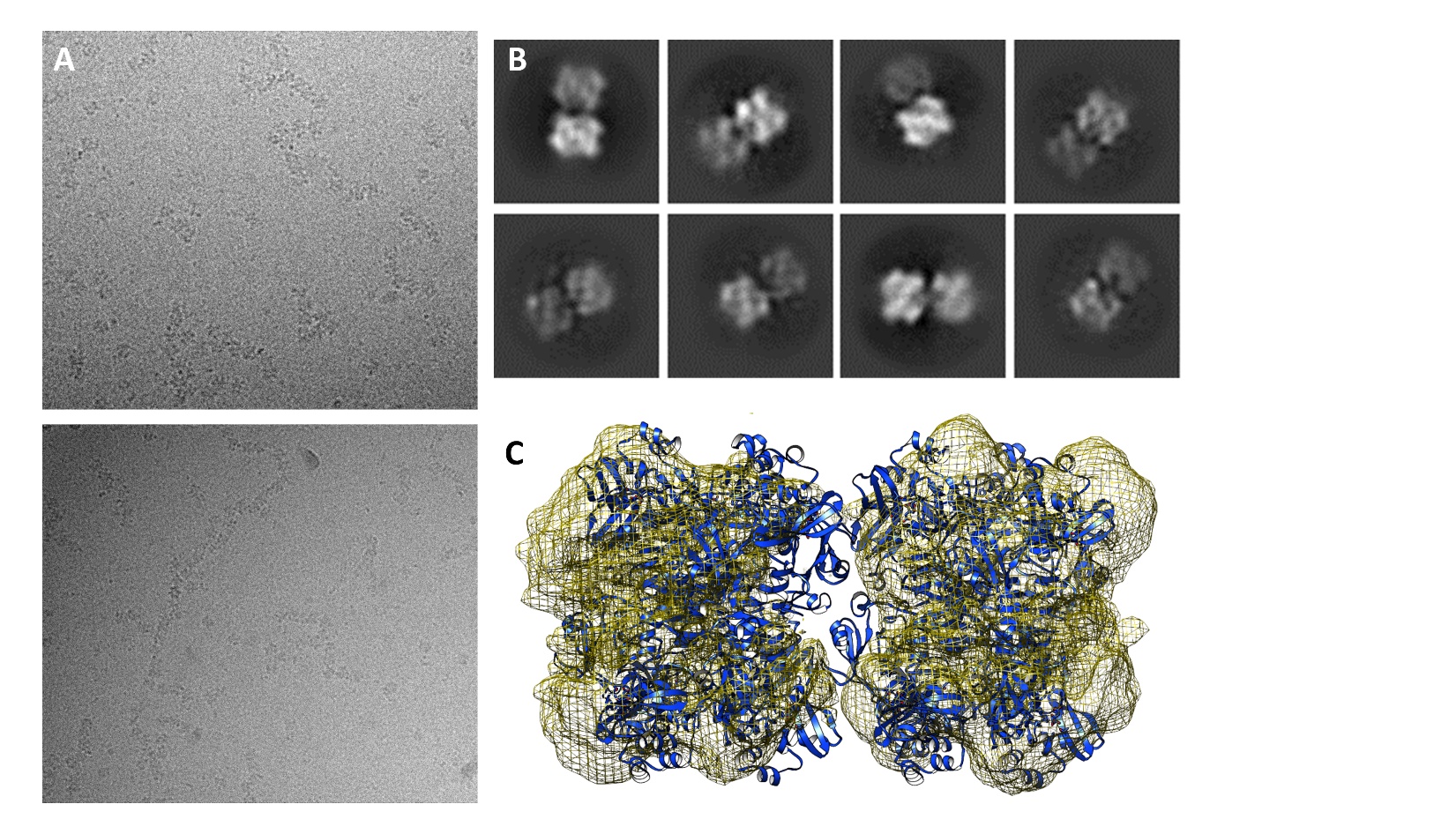


Fig S11. A) 2D crystals formed after adding DNA oligoDT to the sample. B) 2D classes obtained from 2D crystals samples. C) Fourier transformation of 2D crystals images.


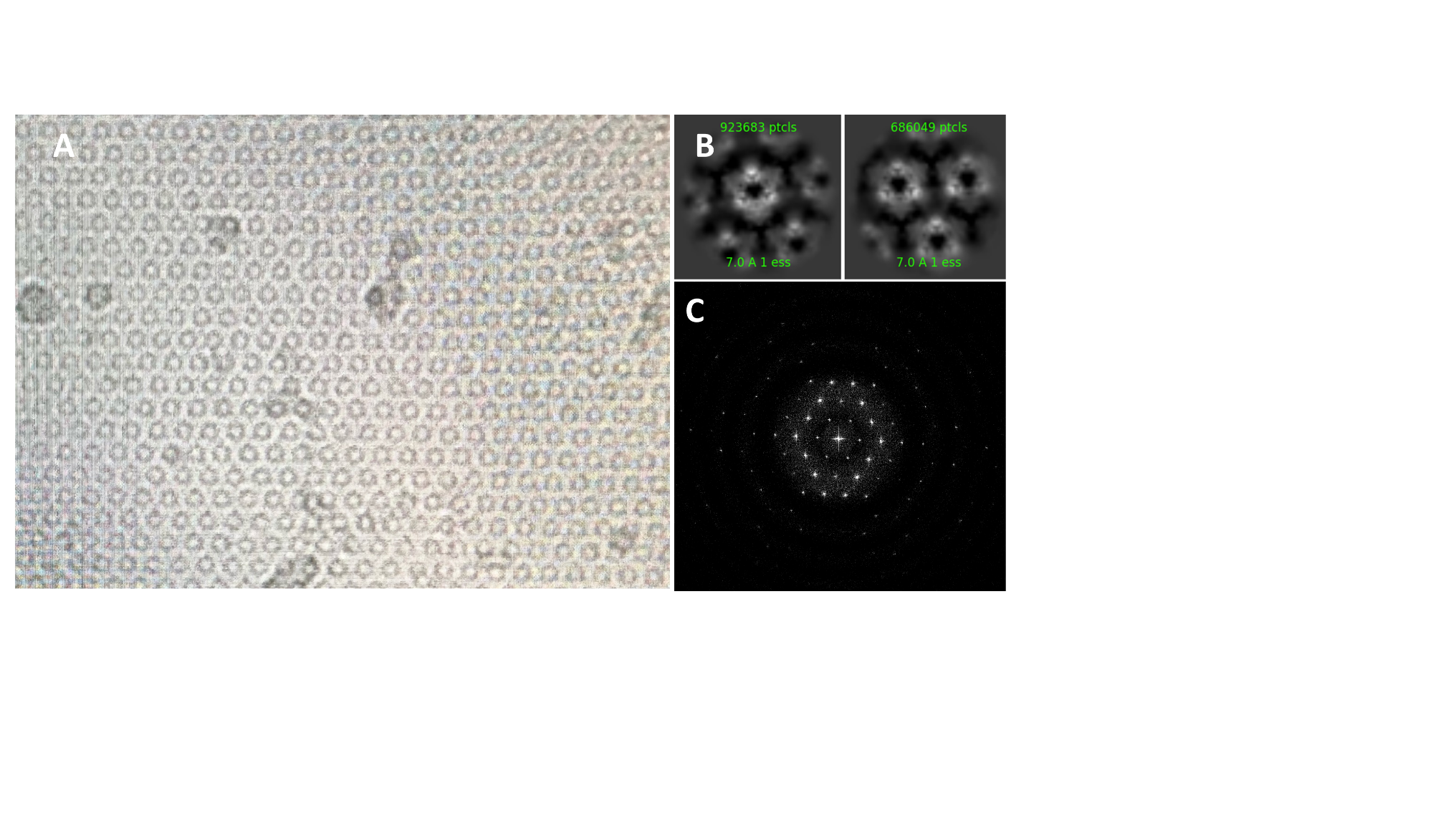


Fig. S12. Different crystals packing between crystals obtained in presence or absence of DNA. A) Crystal packing from crystals obtained without the presence of DNA. B) Crystal packing from crystals obtained in the presence of DNA oligoDT_30_.


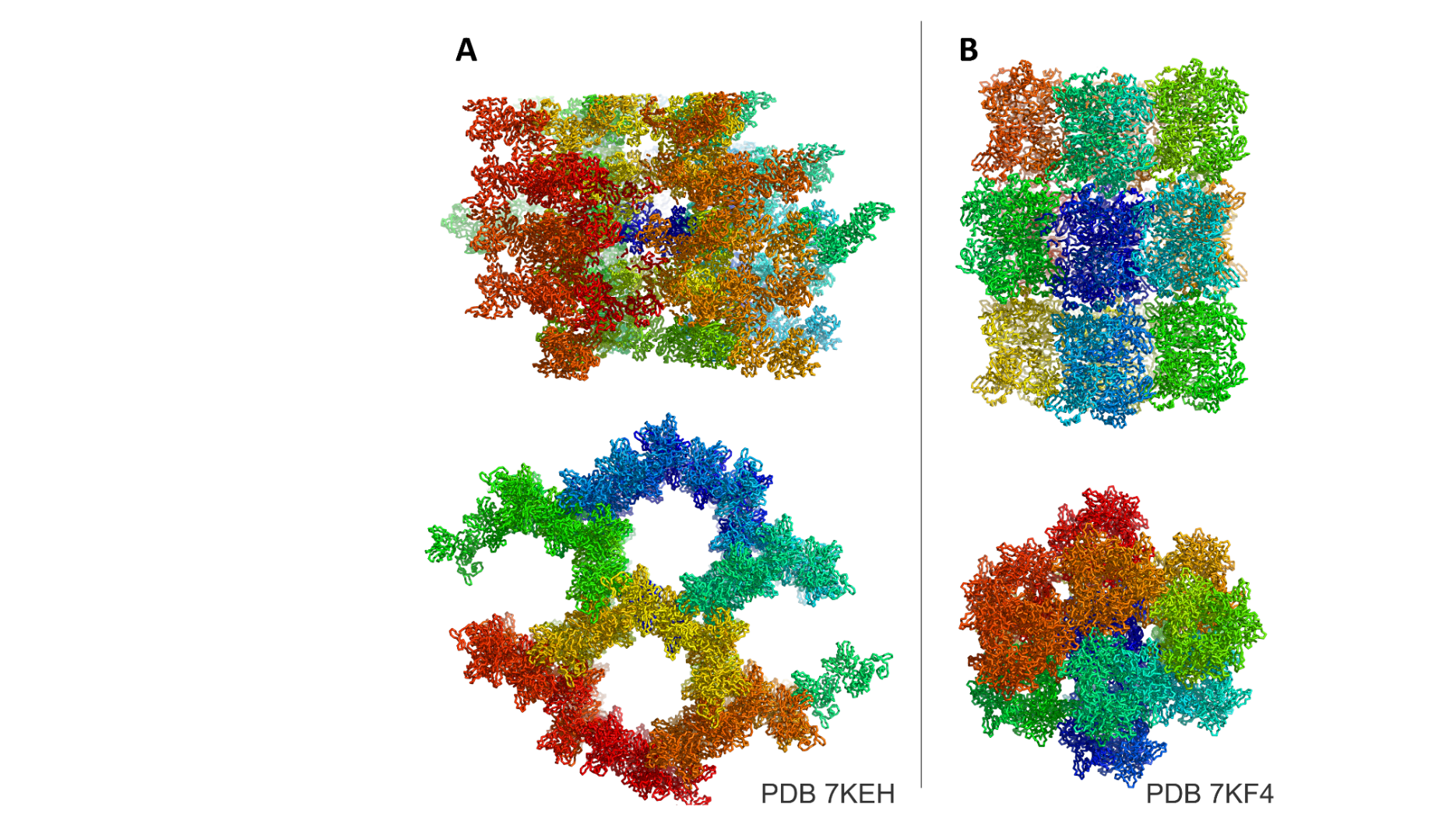


Fig S13. A) Distinct binding modes of NendoU to dsRNA, generated expanding symmetry of PDB 7TJ2. B) Sequence of nsp15 colored according to conservation. C) Surface view of nsp15 structure colored according to conservation. Conservation analyses were performed with Consurf.


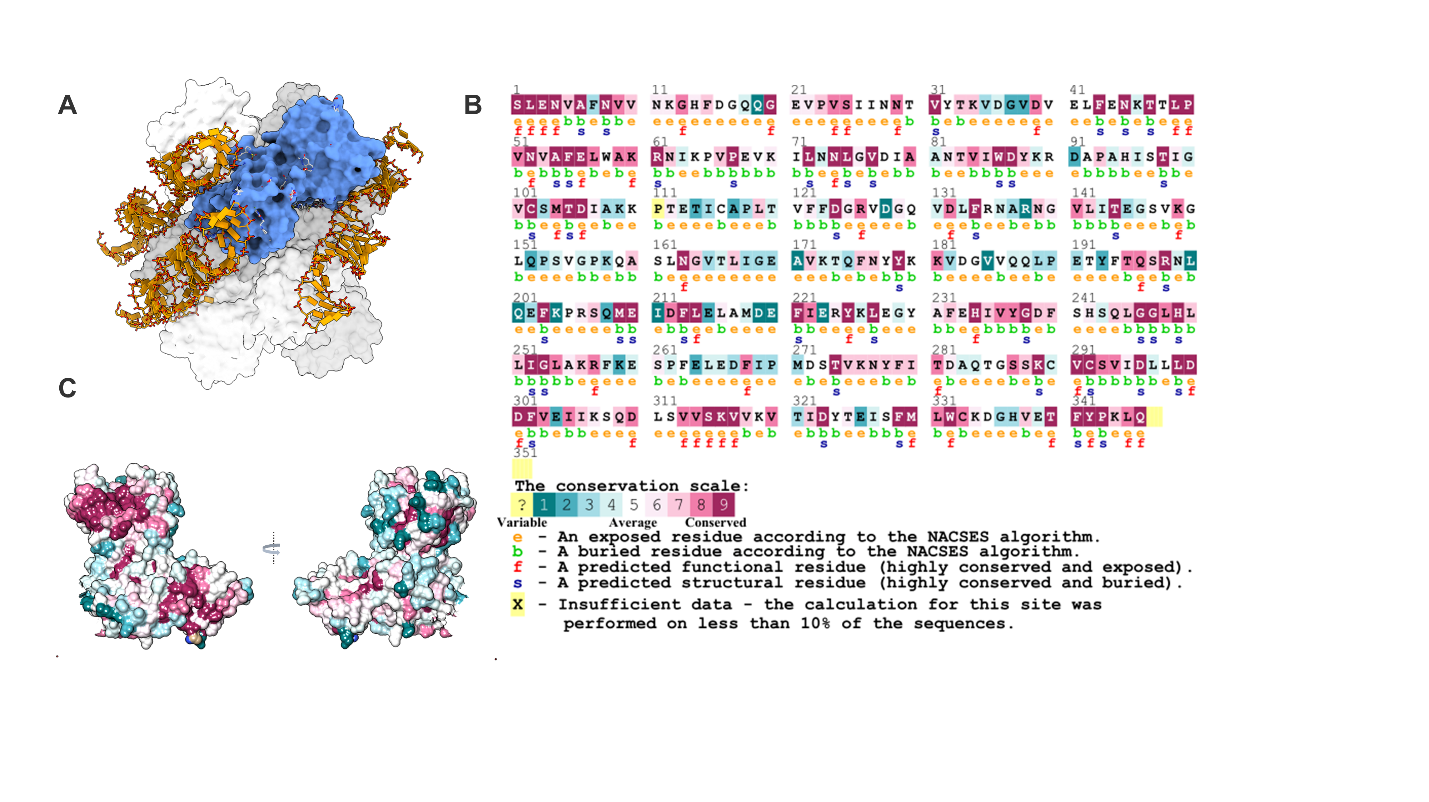


Fig S14. Other fragments identified during fragment screening spread across multiple putative allosteric sites. Fragments are depicted as sticks with yellow carbons, while interacting NendoU residues are depicted as grey carbon sticks.


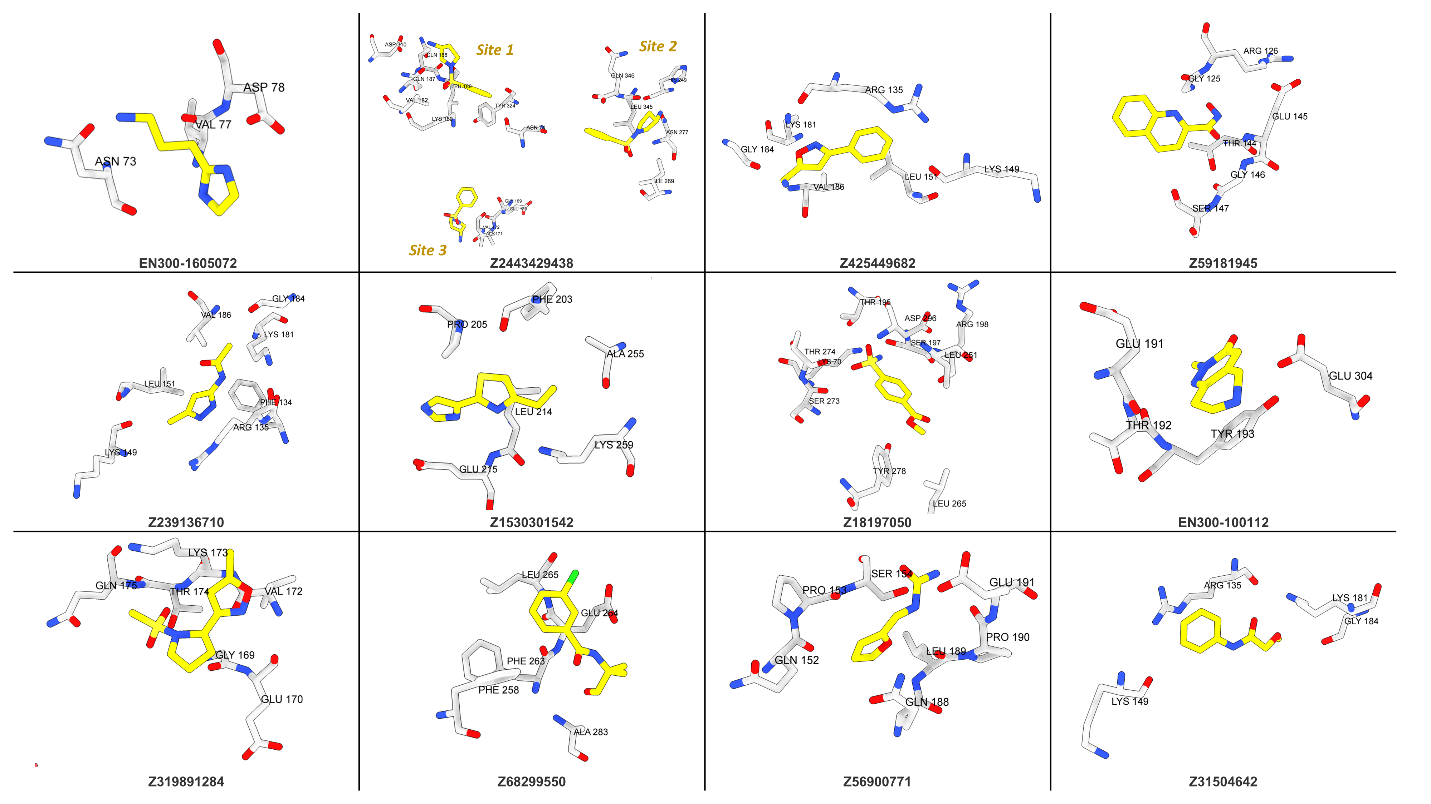


Fig S15. Fragments that caused disruption of active site of one chain. A) View from hexamer colored according to its B-factor (50 to 170 Å2 ), highlighting the oberserved effect on chain A. The highlighted region shows chain A activite site region with one example (dataset #134) of the electron density observed for this region. 2Fo-Fc map is colored in cyan, while Fo-Fc maps are colored in green (positive values) or red (negative values). B) Chemical structure of all fragments identified that caused disruption of chain A active site.


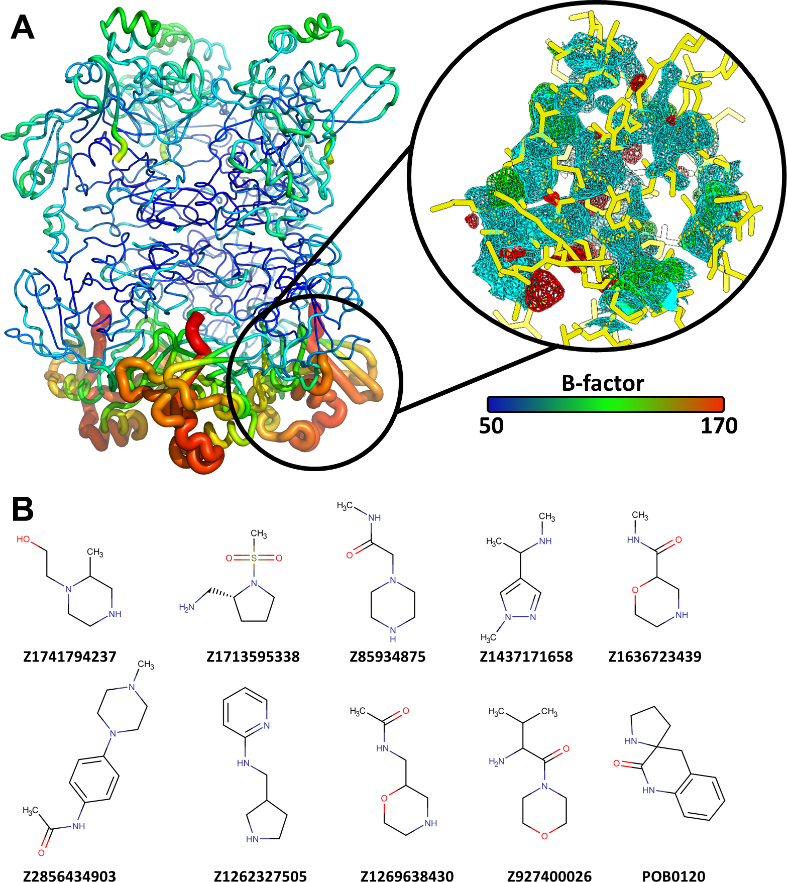


Table S1. Activity profile of NendoU^mon^ and NendoU^hex^ in different buffers. Error is ± SE of fit determined from triplicates.

|  |  |  |  |  | **NendoU^mon^** | | **NendoU^hex^** | |
| --- | --- | --- | --- | --- | --- | --- | --- | --- |
| **Buffer** | **Buffer concentration**  **(mM)** | **NaCL** | **Glycerol (%)** | **pH** | **v_o_ (RFU/min)** | **Error** | **v_o_ (RFU/min)** | **Error** |
|  |  | **(mM)** |  |  |  |  |  |  |
| Sodium acetate | 50 | 150 | 0 | 5.0 | 19.57 | 0.126 | 241.09 | 18.78 |
| Sodium cacodylate (HCl) | 50 | 150 | 0 | 6.0 | 15.49 | 0.078 | 998.43 | 31.25 |
| MES (NaOH) | 50 | 150 | 0 | 6.0 | 16.55 | 0.067 | 92.58 | 2.58 |
| BIS-TRIS (NaOH) | 50 | 150 | 0 | 6.5 | 15.62 | 0.14 | 902.76 | 13.90 |
| Imidazole (HCl) | 50 | 150 | 0 | 7.0 | 2.84 | 0.15 | 804.66 | 3.45 |
| MOPS (NaOH) | 50 | 150 | 0 | 7.2 | 1.42 | 0.09 | 100.27 | 1.62 |
| Tricine (NaOH) | 50 | 150 | 0 | 8.0 | 3.49 | 0.14 | 63.67 | 7.15 |
| TRIS (HCl) | 50 | 150 | 0 | 8.0 | - | - | 174.53 | 5.35 |
| TRIS (HCl) | 50 | 150 | 0 | 8.5 | 2.76 | 0.17 | 43.06 | 2.10 |
| Bicine (NaOH) | 50 | 150 | 0 | 8.3 | 6.00 | 0.24 | - | - |
| HEPES (NaOH) | 50 | 150 | 0 | 7.5 | - | - | 29.74 | 0.81 |
| Glycine (NaOH) | 50 | 150 | 0 | 9.0 | 0.90 | 0.142 | - | - |
| CHES (NaOH) | 50 | 150 | 0 | 9.5 | - | - | 27.56 | 1.36 |
| Sodium borate | 50 | 150 | 0 | 10.0 | - | - | - | - |
| HEPES (NaOH) | 50 | 0 | 0 | 7.5 | - | - | 156.54 | 2.9 |
| HEPES (NaOH) | 50 | 150 | 0 | 7.5 | - | - | 83.65 | 1.20 |
| HEPES (NaOH) | 50 | 500 | 0 | 7.5 | 0.33 | 0.0 | 46.08 | 2.65 |
| HEPES (NaOH) | 50 | 150 | 5 | 7.5 | - | - | 73.07 | 5.03 |
| HEPES (NaOH) | 50 | 150 | 10 | 7.5 | 0.012 | 0.037 | 136.80 | 3.42 |
| PBS | 50 | 150 | 0 | 7.4 | - | - | 74.19 | 2.97 |
| PBS | 50 | 150 | 0 | 6.0 | - | - | 430.43 | 15.01 |

Table S2. Data collection and processing statistics for cryo-EM models of NendoU

|  | **TRIS-HCl pH 7.5** | **BIS-TRIS pH 6.0** | **PBS pH 6.0** |
| --- | --- | --- | --- |
| **Data collection** |  |  |  |
| C2 (µm) | 50 | 70 | 70 |
| Spot size | 6 | 6 | 5 |
| Illuminiation (µm) | 1.1 | 0.99 | 1.25 |
| Magnification | 105k | 105k | 105k |
| Camera | K3 super resolution | K3 super resolution | K3 super resolution |
| Slit width | 20 eV | 20 eV | 20 eV |
| Pixel (physical) (Å) | 0.831 | 0.831 | 0.831 |
| Dose rate (e/px/s) | 14.5 | 15.01 | 18 |
| exposure time (s) | 2.02 | 1.99 | 1.6 |
| total dose (e/Å2) | 42.41 | 43.25 | 41.7 |
| fractions | 40 | 40 | 40 |
| dose per frame (e/Å2/frame) | 1.06 | 1.08 | 1.04 |
| Movies collected | 6,467 | 14,261 | 4,080 |
|  |  |  |  |
| **Model Refinement** |  |  |  |
| Chains | 6 | 6 | 6 |
| Atoms | 16350 | 16386 | 16416 |
| Water | 0 | 0 | 0 |
| Bonds (RMSD) |  |  |  |
| Length (Å) | 0.009 (0) | 0.008 (0) | 0.006 (0) |
| Angles (°) | 0.756 (1) | 0.695 (3) | 0.553 (1) |
| MolProbity score | 1.96 | 1.71 | 1.85 |
| Clash score | 12.75 | 6.92 | 5.72 |
| Ramachandran plot (%) |  |  |  |
| Outliers | 0 | 0 | 0 |
| Allowed | 4.88 | 4.73 | 2.03 |
| Favored | 95.12 | 95.27 | 97.97 |
| Rama-Z (Ramachandran plot Z-score, RMSD) |  |  |  |
| whole (N = 2076) | 1.42 (0.18) | 0.98 (0.19) | 0.22 (0.17) |
| helix (N = 516) | 1.13 (0.20) | 0.83 (0.22) | 0.20 (0.22) |
| sheet (N = 312) | 0.81 (0.28) | 0.54 (0.32) | 0.93 (0.30) |
| loop (N = 1248) | 0.83 (0.18) | 0.52 (0.18) | 0.56 (0.16) |
| Rotamer outliers (%) | 0 | 0 | 4.96 |
| Cβ outliers (%) | 0 | 0 | 0 |
| Peptide plane (%) |  |  |  |
| Cis proline/general | 0.0/0.0 | 0.0/0.0 | 0.0/0.0 |
| Twisted proline/general | 0.0/0.0 | 0.0/0.0 | 0.0/0.0 |
| CaBLAM outliers (%) | 1.55 | 1.60 | 1.02 |
| d FSC model (0/0.143/0.5) | 2.9/3.0/3.3 | 3.1/3.2/3.4 | 2.4/2.5/2.7 |
| Model vs. Data |  |  |  |
| CC (mask) | 0.75 | 0.81 | 0.83 |
| CC (box) | 0.75 | 0.77 | 0.82 |
| CC (peaks) | 0.7 | 0.74 | 0.79 |
| CC (volume) | 0.74 | 0.79 | 0.82 |
| PDB deposition code | 7RB0 | 7RB2 | 7ME0 |
| EMDB deposition code | EMD-24391 | EMD-24392 | EMD-23786 |

Table S3. Data collection and processing statistics for X-ray models of NendoU

|  |  | Dihedral AU | Hexamer AU | Dihedral AU |
| --- | --- | --- | --- | --- |
|  |  |  |  |  |
| Beamline | | MANACA | MAXIV | MAXIV |
| Wavelenght (Å) | | 1.335 | 0.976 | 0.976 |
| Resolution range (Å) | | 45.0 - 2.9 (3.0 - 2.9) | 49.16 - 2.61 (2.7 - 2.6) | 84.6 - 2.6 (2.74 - 2.6) |
| Space group | | P 63 | P 2 21 21 | P 63 |
| Unit cell (a, b & c; Å, angles) | | 150.6 150.6 110.2  90 90 120 | 85.1 151.1 199.2  90 90 90 | 150.9 150.9 111.1  90 90 120 |
| Unique Reflections | | 31369 (3117) | 78886 (7756) | 37814 (1889) |
| Multiplicity | | 10.0 (10.2) | 13.8 (13.9) | 29.8 (30.1) |
| Completeness (%) | | 99.04 (98.67) | 99.73 (99.73) | 94.8 (60.3) |
| Mean I/sigma(I) | | 5.0 (1.0) | 8.24 (0.72) | 7.6 (1.6) |
| Rpim (%) | | 0.188 (0.912) | 0.06367 (0.9967) | 0.19 (0.60) |
| CC1/2 | | 0.96 (0.31) | 0.998 (0.527) | 0.99 (0.37) |
| *Rwork* | | 0.1949 (0.3064) | 0.2331 (0.3987) | 0.19 |
| *Rfree* | | 0.2332 (0.3290) | 0.2573 (0.4120) | 0.22 |
| Number of atoms | |  |  |  |
|  | ligands | 10 | 78 | 48 |
|  | waters | 357 | 429 | 468 |
|  | Protein residues | 696 | 2088 | 696 |
| RMS(bonds) (Å) | | 0 | 14 | 13 |
| RMS(angles) (o) | | 1,78 | 1.69 | 1.76 |
| Ramachandran favored (%) | | 96.68 | 97.50 | 97.25 |
| Ramachandran outliers (%) | | 0 | 0.10 | 0 |
| Clashscore | | 2 | 2.58 | 2.88 |
| Average B-factors (Å²) | |  |  |  |
|  | Macromolecules | 54.18 | 74.33 | 58.10 |
|  | Ligands | 89.87 | 108.38 | 67.35 |
|  | Solvent | 43.54 | 57.70 | 53.28 |
| PDB code | | 7KEG | 7KF4 | 7KEH |

Table S4. Activity profile of NendoU^mon^. Test was performed in BIS-TRIS buffer pH 6.0 and with 100 nM NendoU^mon^. Error is ± SE of determined from triplicates.

|  | v_o_ (RFU/min) | Error |
| --- | --- | --- |
| control | 2.51 | 0.13 |
| 20 mM MnCl2 | 3.42 | 0.08 |
| 10 mM MnCl2 | 3.81 | 0.15 |
| 5 mM MnCl2 | 3.88 | 0.07 |
| 2.5 mM MnCl2 | 4.33 | 0.19 |
| 20 mM EDTA | 5.17 | 0.11 |
| 10 mM EDTA | 6.12 | 0.12 |
| 5 mM EDTA | 6.18 | 0.17 |
| 2.5 mM EDTA | 7.56 | 0.18 |

NMR spectra for **5’-S-Acetyl-5’-deoxy-5’-thiothymidine**


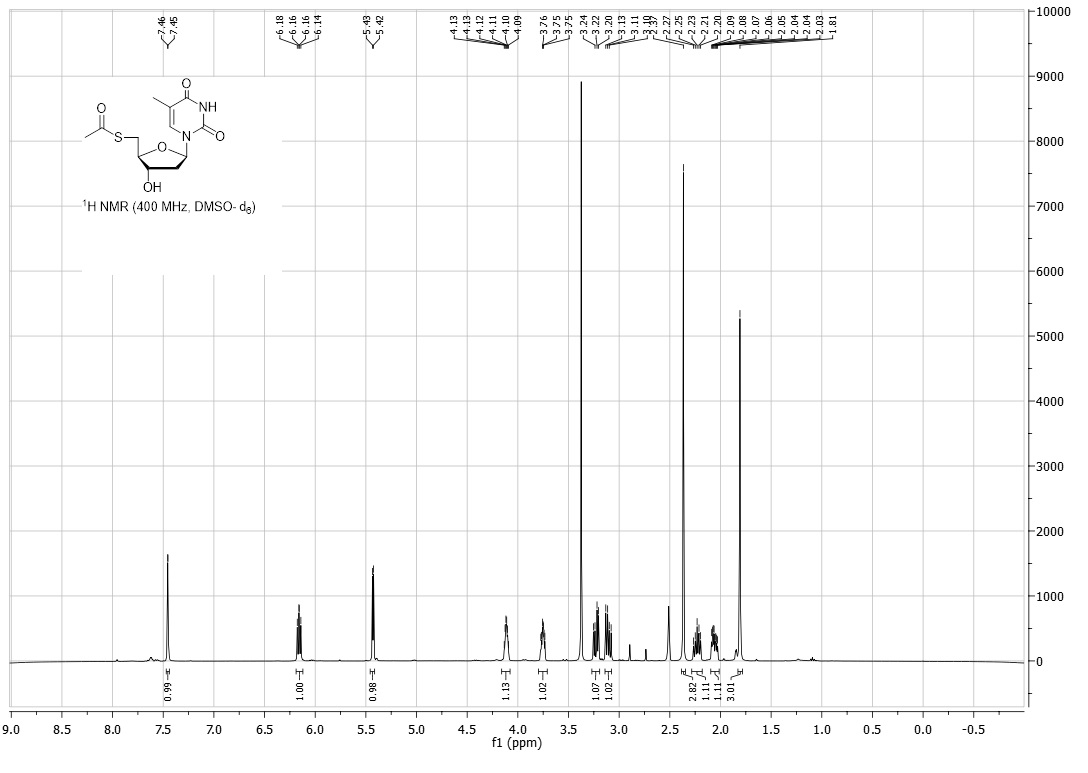


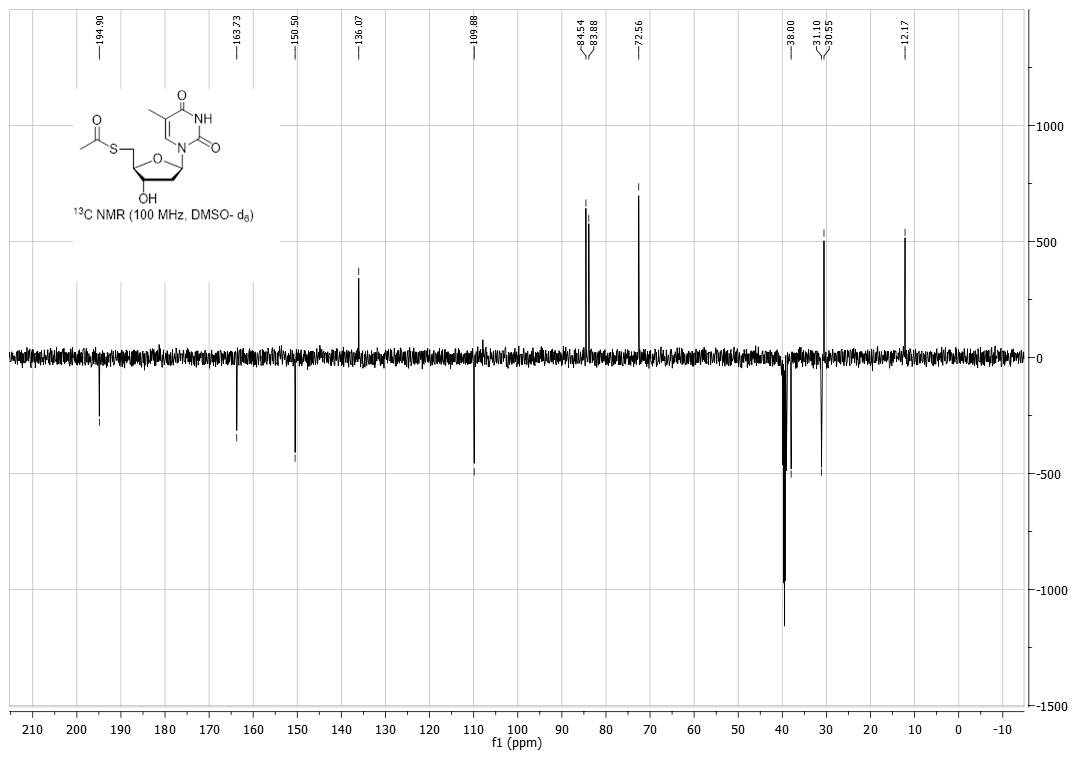


NMR spectra for **5’-deoxy-5’-thiothymidine**


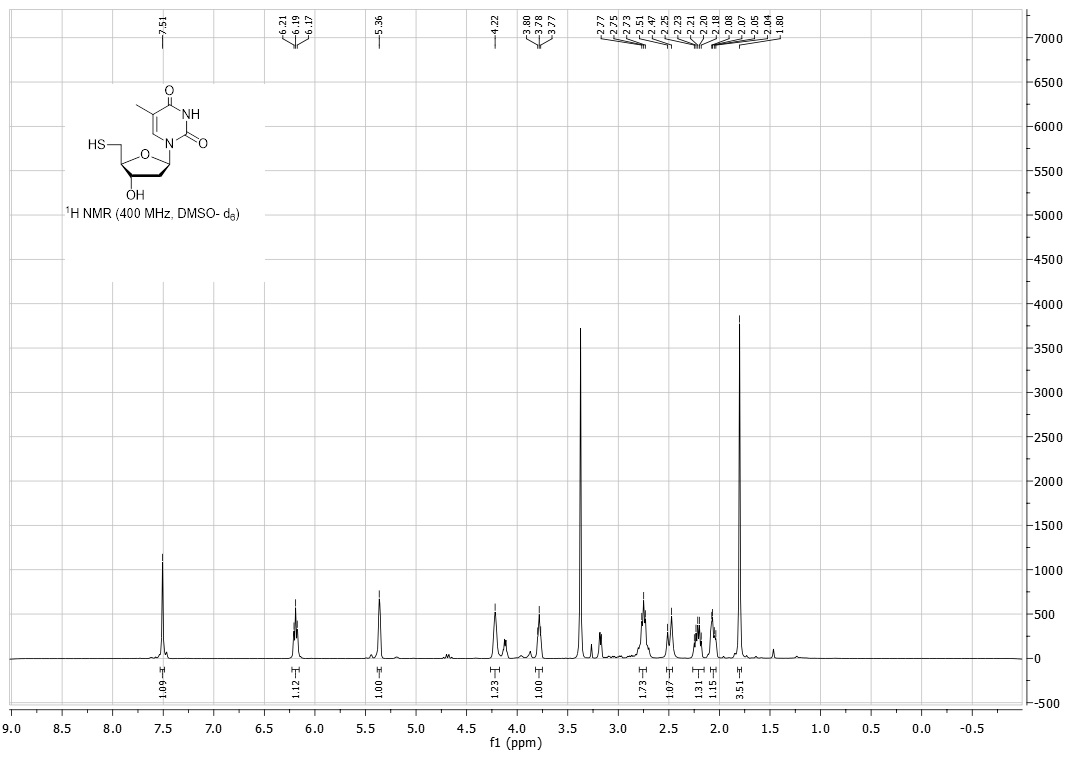


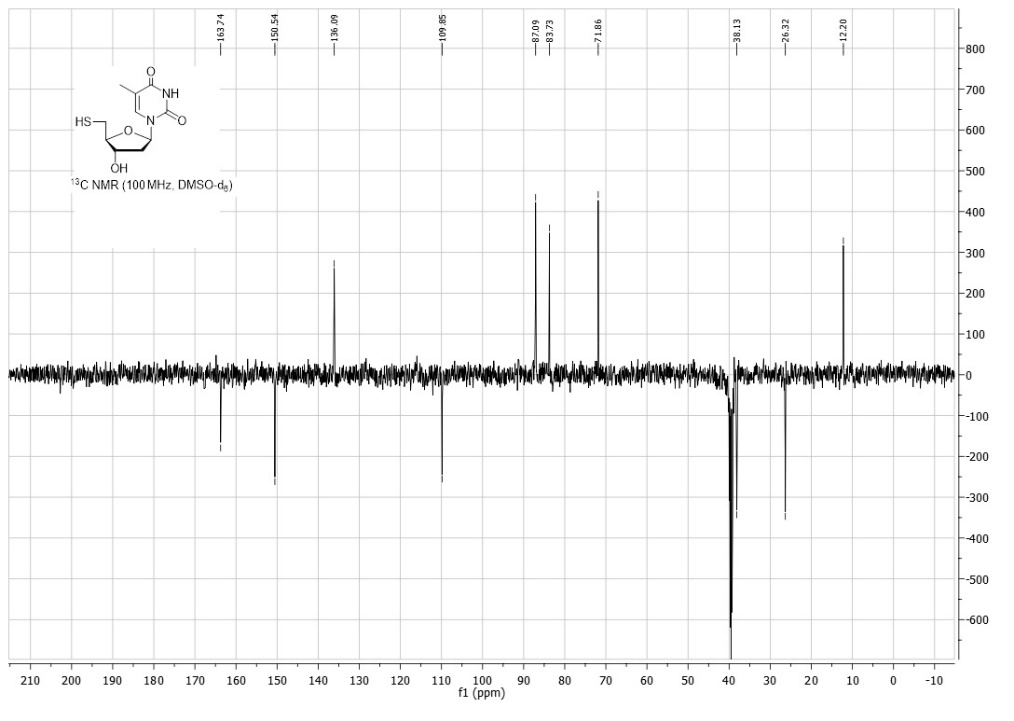

Supplement: gkad314_Supplemental_Files [file gkad314_supplemental_files.zip › SMI-rev.docx]
