# Supplementary material for: Allosteric regulation and crystallographic fragment screening of SARS-CoV-2 NSP15 endoribonuclease
Source: Nucleic Acids Res. 2023 Apr 28;51(10):5255–70. doi: 10.1093/nar/gkad314 (PMC10250223; doi:10.1093/nar/gkad314)
Supplement: gkad314_Supplemental_Files [file gkad314_supplemental_files.zip › Supplementary movie legends.docx]

Movie 1 - 3DVA movement analysis of NendoU at pH 7.5. Movies show three components generated from front and top views.

Movie 2 - 3DVA movement analysis of NendoU at pH 6.0. Movies show three components generated from front and top views.
